# A Comprehensive Review on Phytochemistry and Pharmacological Activities of* Clinacanthus nutans* (Burm.f.) Lindau

**DOI:** 10.1155/2018/9276260

**Published:** 2018-07-04

**Authors:** Leng Wei Khoo, Siew Audrey Kow, Ming Tatt Lee, Chin Ping Tan, Khozirah Shaari, Chau Ling Tham, Faridah Abas

**Affiliations:** ^1^Department of Food Science, Faculty of Food Science and Technology, Universiti Putra Malaysia, 43400 Serdang, Selangor, Malaysia; ^2^Department Biomedical Science, Faculty of Medicine and Health Sciences, Universiti Putra Malaysia, 43400 Serdang, Selangor, Malaysia; ^3^Faculty of Pharmaceutical Sciences, UCSI University Kuala Lumpur Campus, Jalan Menara Gading, UCSI Heights (Taman Connaught), Cheras, 56000 Kuala Lumpur, Malaysia; ^4^Department of Food Technology, Faculty of Food Science and Technology, Universiti Putra Malaysia, 43400 Serdang, Selangor, Malaysia; ^5^Laboratory of Natural Products, Institute of Bioscience, Universiti Putra Malaysia, 43400 Serdang, Selangor, Malaysia

## Abstract

*Clinacanthus nutans *(Burm.f.) Lindau (Acanthaceae), commonly known as Sabah snake grass, is a vegetable and a well-known herb that is considered an alternative medicine for insect bites, skin rashes, herpes infection, inflammation, and cancer and for health benefits. Current review aims to provide a well-tabulated repository of the phytochemical screening, identification and quantification, and the pharmacological information of* C. nutans* according to the experimental design and the plant preparation methods which make it outstanding compared to existing reviews. This review has documented valuable data obtained from all accessible library databases and electronic searches. For the first time we analyzed the presence of flavonoids, triterpenoids, steroids, phytosterols, and glycosides in* C. nutans* based on the results from phytochemical screening which are then further confirmed by conventional phytochemical isolation methods and advanced spectroscopic techniques. Phytochemical quantification further illustrated that* C. nutans *is a good source of phenolics and flavonoids. Pharmacological studies on* C. nutans* revealed that its polar extract could be a promising anti-inflammation, antiviral, anticancer, immune and neuromodulating, and plasmid DNA protective agent; that its semipolar extract could be a promising antiviral, anticancer, and wound healing agent; and that its nonpolar extract could be an excellent anticancer agent.

## 1. Introduction

Utilizing plants as a source of medicines has been practiced for a long time, especially in developing countries where drugs are usually inaccessible or costly, obligating people to use traditional remedies. Vast development in investigating the herbal plants as alternative therapeutic agents for different type of diseases is notable, starting from the late 1980s and continuing in recent years. Malaysia, a country with rich biodiversity, has been gifted with valuable medicinal plants resources in its tropical rainforest. Among them, Acanthaceae is considered as one of the leading families of the dicotyledonous flowering plants and consist of 250 genera and approximately 2500 species [1]. This family is mainly distributed in Indonesia, Malaysia, Africa, Brazil, and Central America and possesses numerous high medicinal value species [2, 3].

*Clinacanthus nutans *(Burm.f.) Lindau, which belongs to the Acanthaceae family, is a very well-known traditional herb and vegetable in Southeast Asia countries and has been chosen for this review study [4]. It has a few vernacular names such as “belalai gajah” or “pokok stawa ular” (Malay), “e zui hua” or “you dun cao” (Chinese), “payayor” or “slaed pang pon” (Thai), and “daun dandang gendis” or “kitajam” (Javanese). Since a long time ago, this plant is famous as an antidote for snake bites, herpes infection, skin infection, cancer, burns and scalds, dysentery, and diabetes in Thailand, Indonesia, and Malaysia [5–8]. Numerous studies on this plant have been published by researchers from Thailand since 1967. Since 1987,* C. nutans* has become one of the herbs recommended for use in hospitals and was included in the Primary Health Care Programme by the Government of Thailand [9, 10]. The most used part of the plant was the leaf, and the common prescription method was decocting the plant with water for oral ingestion or soaking it in the alcohol for a week for topical application to an affected area [11]. In 2011, this plant began gaining popularity in Malaysia, and various types of* C. nutans* related studies have been conducted by Malaysian scientists.

Authors noticed the publication of reviews on this plant, where some of them also discussed the topics chosen in this paper [2, 12–16]. However, authors target to provide a well-managed summary and possible recommendation on the existing studies so that the readers could acquire much information regarding the current and future direction of this plant from present review just by a glance. Thus, a well-organized and critical review paper on this plant is in demand to reduce redundant studies and allow new insight into the research direction to be made. Therefore, instead of discussing a few selected studies, present review discusses and covers thoroughly the previous* C. nutans* studies related to the topics chosen. The details such as pre- and postharvesting preparation methods of the plant extract as well as the experiment design applied in the studies of phytochemical screening, identification and quantification, and pharmacological activities of* C. nutans* are systematically categorized, compared, and summarized. Information on the preharvesting (plant age and plant origin) and postharvesting (plant part used, drying method, extraction method, extraction solvent, and storage duration) methods of the plant as well as on the experiment design employed was considered and was emphasized in the discussion section which makes present review different from the others. Different plant preparation methods and experimental designs will result in the generation of different conclusions, even for the same plant. Thus, including and correlating all variable factors and parameters in the discussion will help to determine the pro and cons of each study and lead to a better summary in the end. We hypothesized that, through all these efforts, a good summary, precautionary step, challenge, limitation, new idea, or a clearer future perspective may be initiated.

## 2. Phytochemistry

### 2.1. Phytochemical Screening

For the last few decades, the study of plants has progressed rapidly [17]. Plants contain both primary and secondary metabolites. Primary metabolites are those chemical constituents that are inherently present in most organisms and have a direct involvement in plant growth [18]. On the other hand, secondary metabolites are the bioactive phytochemicals that are normally produced by a plant in response to specific environmental stresses and that are considered to possess additional health benefits [18]. Throughout the years, the common practice of researchers was started with the screening of plant. Phytochemical screening tests, thin layer chromatography (TLC) and Fourier transform infrared spectroscopy (FTIR) are among the most popular methods used as they are easy to conduct, of low cost, and time-effective. These methods are able to provide guidelines on the class and functional groups of the chemical constituents that are present in the plant.

Table 1 lists the occurrence of chemical classes in* C. nutans* extract, as determined through specific phytochemical screening tests. The phytochemical screening tests adopted include Mayer's reagent, modified Dragendorff's reagent, or the Wagner test for alkaloid detection; the frothing test for saponin, the alkaline reagent, Shinoda, or lead acetate tests for flavonoids; the frothing, ferric chloride, potassium dichromate, or gelatin tests for tannins; the copper acetate test for diterpenes; Salkowski's test, or Liebermann-Burchard test for phytosterols; the iron (III) chloride test for phenolic compounds; the pseudoindicans test for iridoids; Grignard reagent, Baljet, Keller-Kiliani, or Molisch tests for glycosides; Molisch or Fehling tests for carbohydrates; Salkowski's test for steroids and sterols; and the biuret test for proteins and amino acids [6, 19–22]. As a summary, flavonoids, triterpenoids, steroids, phytosterols, and glycosides are the phytochemical classes that are most likely present in* C. nutans* extract when the plant is extracted by a polar solvent, such as water, methanol, ethanol, or an aqueous organic solvents or by a semipolar (e.g., chloroform) solvent. The presence of alkaloids, saponins, and tannins in* C. nutans* depends on the plant origin and the postharvesting method used. For instance, alkaloids were typically present in water and chloroform leaf extracts and were absent in methanol and aqueous methanol leaf extracts collected from Malaysia. By contrast, saponins were present in methanol extract cultivated in Malaysia and absent in water extract from Indonesia and chloroform and aqueous methanol extracts from Malaysia. Tannins were found in leaves extracted with water, aqueous methanol, and chloroform, but not those extracted with methanol. For the presence of quinones, iridoids, carbohydrates and protein, and amino acid, further confirmation is required as there has been only one study conducted on each of them to date.

In 2012, Roeslan and his teams [11] compared leaves from two different sources, Thailand and Indonesia, using TLC separation method. Their findings showed that there was a quality difference and indicated that the quality of the sample from Indonesia was better, as this sample was thicker than the sample from Thailand. Chelyn et al. [23] used the HPTLC method to screen for the presence of* C*-glycosyl flavones in three* C. nutans* plant samples that underwent the same postharvesting processing but that were from three different geographical locations in Malaysia: Taiping (Perak), Kota Tinggi (Johor), and Sendayan (Negeri Sembilan). All the results indicated that schaftoside was present in* C. nutans* collected from all the investigated locations, whereas isoorientin, isovitexin, orientin, and vitexin were found only in the plants harvested from Perak and Johor, not in the sample harvested from Negeri Sembilan. Tiew et al. [24] used UV-visible and FTIR spectrophotometry for the functional group identification of* C. nutans* leaves dried under the shade and macerated with methanol. Based on the results obtained, the extract showed two major bands 240–400 nm and the presence of an -OH band and a C=O stretch suggests that the constituents in the extract have -OH group and C=O functional groups.

### 2.2. Phytochemical Identification

Phytochemical screening methods can only provide a clue of the chemical class present in the plant. To further validate the identity of the compounds, column chromatography (isolation) and TLC (separation and purity) together with nuclear magnetic resonance (NMR) spectroscopy (identification and structural elucidation) are the most conventionally used techniques. In addition, the emergence of advanced analytical tools such as liquid chromatography mass spectrometry (LCMS), gas chromatography mass spectrometry (GCMS), and NMR spectroscopy provides researchers with useful alternatives for identification.

#### 2.2.1. Phytochemical Identification Using Column Chromatography-Spectroscopy Analysis

A summary of the phytochemicals that have been identified in this plant is provided in Tables 2(a) and 2(b).  Table 2(a) displays those compounds that have been isolated through column chromatography and identified using spectroscopic analysis, the results from which were tabulated according to the phytochemical class. To date, the pure compounds that have been isolated from* C. nutans* include 2 triterpenoids; 6 phytosterols; 8 phenolics, including 7* C*-glycosyl flavones and 1 phenolic acid; 5 sulfur-containing glucosides; 8 sulfur-containing compounds; 7 lipid related compounds; and 8 chlorophyll derivatives.

#### 2.2.2. Phytochemical Identification Using Advanced Spectrometric and Spectroscopic Analysis

Meanwhile, Table 2(b) shows a list of compounds identified according to the type of the advanced spectrometric and spectroscopic analytical tools used. There are a total of 6 research teams that have used GCMS for compound identification [19, 28–32], 4 research teams that have used LCMS analytical tool [4, 19, 30, 33], and 1 research team that has used proton and* J-*resolved NMR approaches [4]. Based on GCMS identification results, Cheong et al. [29] found a rich variety of triterpenoids and phytosterols in the extract. Yong et al. [32] showed that of the 14 phytochemicals identified in the chloroform extract, 1,2-benzenedicarboxylic acid, mono(2-ethylhexyl)ester, a common plasticizer, was the most abundant compound. Mustapa et al. [30] found that phytol was the main compound in the microwave (MAE) and Soxhlet assisted extracts, while palmitic acid was dominant in the supercritical fluid assisted (SFE) extract. A study by Abdul Rahim et al. [19] identified a total of 39 peaks, but only 8 major compounds have been listed in Table 2(b). In addition, Alam et al. [28] have successfully identified a total of 48 compounds, the most abundant of which were neophytadiene, glycolic acid, phosphonic acid, catechol, butanedioic acid, vanillic acid, gallic acid, pinitol, phytol, squalene, tocopherols, and campesterol. Moreover, Teoh et al. [31] have found that the ethyl acetate root extract possessed more compounds than the methanol root extract. Among the compounds identified, lupeol was the main compound present in both extracts.

Using LCMS in negative ionization mode, Huang et al. [33] successfully identified 6* C*-glycosyl flavones, the research team of Khoo et al. [4] was able to identify 8 compounds, Mustapa et al. [30] team have found 10 phenolic compounds from the aqueous ethanolic extract, and Abdul Rahim et al. [19] have detected 16 phenolic compounds. Khoo et al. [4] have also tentatively identified 20 primary metabolites and 22 secondary metabolites in 70% ethanol extract through proton and* J-*resolved NMR spectroscopy.

### 2.3. Phytochemical and Nutrient Content

Environmental condition, geographical location, cultivation practices, genetics, pre- and postharvesting methods practiced and some other unexpected factors will cause phytochemical level variations in a plant even in the same species [18]. Thus, this section reveals the phytochemical content of* C. nutans* including the total phenolic content (TPC) in Table 3, the total flavonoid content (TFC) in Table 4, and the nutritional composition of* C. nutans* in Table 5. Generally, the phytochemical content of* C. nutans* has been evaluated using simple chemical assay or high performance liquid chromatography (HPLC) methods. Compared to other tests, determination of TPC and TFC is preferred due to the interest in the therapeutic benefit imparted by phenolics and flavonoid; in addition, they are easy, time-effective, and cost-effective and well-established methods.

The Folin-Ciocalteu method is the most established method for determining the total phenolic content of* C. nutans* and gallic acid or, in some cases, tannic acids are the most common standards used for TPC calibration. Values are normally expressed in mg gallic acid equivalent (GAE)/g dry weight (DW) or dry material (DM). Total phenolic content is based on a colorimetric reaction between easily oxidized polyphenols or hydroxylated aromatic compounds and phosphotungsten-polymolybdic acid [34]. The redox reaction results in the formation of blue chromophores consisting of phosphotungstic-phosphomolybdenum complexes, which have a maximum absorption at 765 nm and are proportional to the total quantity of phenolic compounds [34]. Among the TPC tests conducted, the chloroform fraction prepared by Hamid and Yahaya [35] from a Malaysian farm that underwent sonication was detected to have the highest TPC, with 119.29 ± 0.07 mg GAE/g DW. In addition, Lusia Barek et al. [36] also illustrated that an unfermented leaf subjected to drying in a microwave oven and infusion in water for 20 min retained a high TPC value of 177.80 ± 19.10 mg tannic acid equivalent (TAE)/L.

The flavonoid level in* C. nutans* was mostly determined by the aluminum chloride (AlCl_3_) method. Essentially, the detection of flavonoid (the colored complexes) is conducted using the reaction between AlCl_3_ and the carbonyl and hydroxyl groups of flavonoids in an alkaline solution [18]. As shown by the results in Table 4, TFC was determined using quercetin as the positive control and the results were expressed in mg quercetin equivalent (QE)/g dry weight (DW) sample, although there were also some studies that used catechin, rutin, or butylated hydroxytoluene (BHT) as the positive control. Again, the chloroform fraction of* C. nutans* that underwent sonicated extraction was able to retain more TFC (937.67 ± 0.02 mg BHTE/g) than those undergoing other combination of pre- and postharvesting methods [35].

Other phytochemical content studies on* C. nutans* included hydroxycinnamic acid content determination using Arnow's reagent. The results suggested that oven-dried and hot water extracted* C. nutans* extract possessed 0.91 ± 0.01 mg caffeic acid equivalent/g dry material (mg CAE/g DW) [37]. For the chlorophyll content of* C. nutans*, Raya et al. [38] found that, compared to mature stem (32.27 mg/100 g) or plant sample that kept for 4 days (17.97 mg/100 g), a young leaf or plant sample subjected to 1 day of storage possessed a higher chlorophyll content with 64.35 mg/100 g and 78.83 mg/100 g, respectively. By contrast, Mustapa et al. [30] found that the absolute ethanol aerial extract of* C. nutans* possessed a higher chlorophyll content (1.30 g/g DM) than absolute acetone aerial extract (1.06 g/g DM). On the other hand, Raya et al. [38] showed that* C. nutans* leaves harvested at a young stage had a higher content of ascorbic acid (0.38250 mg/100 g). The results also revealed that increasing the storage duration from 1 day to 4 days caused a reduction in the ascorbic content of the extract from 0.49833 mg/100 g to 0.23083 mg/100 g. For phytosterol determination using Liebermann-Burchard reagent, one study showed that increasing the organic solvent ratio in the solvent mixture will result in a decrease in the phytosterol content [30]. This study also suggested that the supercritical fluid extraction (SFE) method allowed higher phytosterol retention (1.35 ± 0.12 mg of *β*-sitosterol (BS)/g DM), followed by pressurized-microwave assisted extraction (p-MAE) for the 86% ethanolic extract (1.19 ± 0.22 mg BS/g DM), microwave assisted extraction (MAE) of the 86% ethanolic extract (0.70 ± 0.10 mg BS/g DM), and Soxhlet extraction of the absolute ethanolic extract (0.47 ± 0.20 mg BS/g DM). Additionally, the nutrient composition of* C. nutans* has also been determined by Sarega et al. [39] and Yu et al. [40] through proximate, vitamin, and mineral analysis as shown in Table 5.

Meanwhile, there were also studies that used HPLC for the quantification of phenolic compounds [23, 39, 41–43] and, in the case of Mustapa et al. [30], for the quantification of a phytosterol compound, *β*-sitosterol. The HPLC parameters used and the sample details are tabulated into Table 6. Although Wong et al. [43] attempted to quantify 4 phenolics compounds, only quercetin was found (0.25 ± 0.01 *μ*g per mg of dried sample), whereas Chelyn et al. [23] quantified schaftoside, orientin, isovitexin, and vitexin content for* C. nutans* leaves collected from 3 different sources. The results revealed that* C. nutans* collected from Taiping, Perak, Malaysia, possessed the highest amount of all 4 phenolics: 17.43 ± 0.01 mmol/g of schaftoside, 0.86 ± 0.04 mmol/g of orientin, 2.01 ± 0.02 mmol/g of isovitexin, and 0.91 ± 0.03 mmol/g of vitexin. Ghasemzadeh et al. [41] found that samples with different maturity and from different plant parts retained different phenolics and flavonoids. Six-month-old bud extract was found to be able to retain the highest concentration of gallic acid (5.96 ± 0.55 mg/g DW). Sarega et al. [39, 42] analyzed and quantified a total of 8 phenolics in the extract. Both studies found that protocatechuic acid was the most prominent phenolic acid in the hot water, water, and 80% methanol leaf extracts (33.28 ± 0.01 mg/g extract). Finally, aerial extract that underwent SFE possessed the highest amount of *β*-sitosterol (0.83 ± 0.10 mg/g DM) followed by the 86% ethanolic p-MAE extract (0.65 ± 0.14 mg/g DM), 86% ethanolic MAE extract (0.52 ± 0.10 mg/g DM), and Soxhlet assisted extract (0.23 ± 0.18 mg/g DM) [30].

## 3. Pharmacological Activity

### 3.1. Pharmacological Activity of* C. nutans* Extracts and Fractions

A number of* C. nutans*' traditional uses have been further verified by laboratory experiments. They have been categorized into antivenom, analgesic, anti-inflammatory, immunomodulating, neuroprotective and neuromodulating function, antidiabetic and *α*-glucosidase inhibitory, antioxidant, antiviral, antibacterial, antifungal, anticancer, wound healing, plasmid DNA protective, lipid elevated inhibition, and oral mucositis and stomatitis treatment activities. Tables 7(a–o) summarize all the available data regarding the pharmacological activities of* C. nutans* extracts and fractions accordingly. All the tables were systematized based on experimental model, followed by type of assay and polarity of extract used. Generally, diverse results can be observed for the same pharmacological activity. The extract preparation method, origin, concentration, and assays type are the primary factors that contributed to the variation in results, and a short summary and point of view have been included in each section.

#### 3.1.1. Antivenom Activity

Table 7(a) summarizes the antivenom tests that have been carried out thus far on* C. nutans.* The experiments examined the antivenom properties of* C. nutans*' leaves against snake, scorpion, and bee venoms. There were a total of 2* in vitro* and 4* in vivo* antivenom studies, and the extracts tested were prepared from water, ethanol, and aqueous ethanol. From the results of the* in vitro* studies, the water extract at 0.706 mg/mL exhibited a moderate anti-scorpion venom effect through a direct inactivation mechanism [65] while the aqueous ethanol extracts were ineffective against bee venom [66]. On the other hand, in 3 out of the 4* in vivo* studies conducted, the extracts (2 water and 1 aqueous ethanol extracts) notably did not exhibit antivenom effects after the test subjects were given different doses (6 to 2000 mg/kg bw) of the extracts through intraperitoneal injection (i.p.), intervenous injection (i.v.), or per os (p.o.) [5, 7, 67]. Although there was 1* in vivo* study proposing that the water extract of* C. nutans* could exhibit a moderate anti-snake venom effect, the study did not include the administration method or the doses given [67]. Thus, defining a suitable extract that is biologically active as an antidote for snake and insect venoms is ambiguous. In summary, among the extracts tested, only water leaf extract exhibited potential* in vitro *inhibition effect towards snake and insect venoms via direct inactivation mechanism. Further animal studies that focus on studying the metabolites of water extract and their reaction with the mediators that responsible for the direct inactivation pathway are worth emphasizing. Clinical study that investigates the efficacy of water extract as an antivenom agent through application of topical formulation on the patients might lead to a possible breakthrough.

#### 3.1.2. Analgesic Activity

Table 7(b) displays the scientific studies that investigated the analgesic ability of* C. nutans* through 3 different types of* in vivo* assays: the acetic acid-induced writhing test, formalin-induced paw licking test, and hot plate test. Three studies have used the acetic acid-induced writhing test to examine the analgesic effect of* C. nutans* extracts prepared using 4 different types of solvents [19, 68, 69]. The results suggested that treating mice with the* n*-butanol extract at 90 mg/kg as well as with the methanol extract at 279.3 mg/kg was as potent in terms of analgesic properties as treating them with phenylbutazone at 100 mg/kg. On the other hand, the study by Abdul Rahim et al. [19] was the only one that used formalin-induced paw licking test to evaluate the analgesic properties of* C. nutans*. The results suggested that the methanol extract, with a half maximal effective concentration (EC_50_) at 227.7 mg/kg, was able to relieve pain in the late phase (centrally inflammatory induced pain pathway) via opioid/nitric oxide- (NO-) mediated, but cyclic guanosine monophosphate- (cGMP-) independent modulation systems. In addition, the analgesic effects of the absolute methanol, ethanol, and* n*-butanol extracts have been examined through the hot plate test. The results suggested that 500 mg/kg methanol extract was effective in alleviating the pain response at the interval from 60 to 210 min while both the ethanol and butanol extracts showed no analgesic effects up to 5 g/kg. Overall, methanol extract of* C. nutans* exerted potential analgesic activity in both acute and persistent pain tests. The study also suggested that the presence of phenolics particularly gallic acid, caffeic acid, ferulic acid, vitexin, and apigenin which previously reported to exert antinociceptive activity which might be the main activity contributors. Since methanol extract might be a potential analgesic agent, it is important to further investigate the efficacy dosages, sites of action, and the level of the extract to modulate the pain along the pathway suggested. Furthermore, study may also provide information regarding antinociception properties of* C. nutans* methanol extract on visceral and neuropathic pains.

#### 3.1.3. Anti-Inflammatory Activity

Table 7(c) summarizes the anti-inflammatory effect of* C. nutans.* The anti-inflammatory effect of* C. nutans* was assessed based on* in vitro* assays, such as the macrophage activator* N*-formyl-methionyl-leucyl-phenylalanine- (fMLP-) induced neutrophil elastase release superoxide anion generation, lipopolysaccharides (LPS) induced toll like receptor 4 (TLR-4), NO Griess, and cytokine production assays. Furthermore, the types of* in vivo* experiments used to evaluate the acute anti-inflammatory properties of* C. nutans* were the acetic acid-induced vascular permeability model and ethyl phenylpropiolate- (EPP-) induced rat ear oedema and carrageenan-induced paw oedema model, whereas its subchronic anti-inflammatory effect was assessed using the granuloma pouch model, which mimics subchronic inflammation in humans. For the* in vitro* anti-inflammatory effects, both the methanol and 80% ethanol extracts have been evaluated through the neutrophil elastase release and superoxide anion generation assays [52, 70]. The results showed that 10 *μ*g/mL of 80% ethanol extract inhibited 68.33% elastase release, which was more effective than the methanol extract (<20%), while to inhibit superoxide radical formation, both extracts at 10 *μ*g/mL showed comparable efficacy, as they inhibited approximately 30% superoxide formation [52, 70]. For the LPS-induced TLR-4, NO, and cytokine production assays, the results suggested that the polar (methanol and dichloromethane) extract possessed a better IC_50_ value (<22 *μ*g/mL) for inhibiting TLR-4, NO and proinflammatory cytokine production than the nonpolar (hexane and diethyl ether) extract [62]. For the* in vivo* acute anti-inflammatory properties of* C. nutans*, the most effective dose was pretreatment of 9 mg EPP/ear of methanol extract on the rat which resulted in 79% oedema inhibition at 15 min and 44.4% myeloperoxidase (MPO) reduction after 120 min of induction [70].

Additionally, in a comparison of the methanol, ethanol, and* n*-butanol extracts, a 1 h pretreatment of 200 mg/kg methanol extract administered via p.o. to carrageenan-induced rats was found to inhibit 59% oedema formation [70]. The acetic acid-induced vascular permeability model showed that, among the water, methanol, chloroform, and* n*-butanol extracts, the butanol extract at 540 mg/kg was superior at exhibiting an anti-inflammatory effect [68, 69]. Ethanol, aqueous ethanol,* n*-butanol, and cold creams have been evaluated for their subchronic anti-inflammation properties. The results showed that application of 125 mg of* C. nutans* cold cream to a rat topically throughout the experimental period inhibited granuloma formation by as much as 50.98%, which is comparable to the inhibitory effect of 0.25% prednisolone (56.82%) [71]. According to the previous* C. nutans* anti-inflammatory studies, the extract prepared from polar solvent notably exhibited a promising anti-inflammatory properties* in vitro *(at a dose less than 30 *μ*g/mL) and* in vivo* (at a dose less than 300 mg/kg). Therefore, it is greatly desired to focus on the relationship between the mode of action of* C. nutans *polar extract in both biological and nonbiological anti-inflammatory systems. In addition, since polar extract exhibited potential anti-inflammatory activity, knowledge of extract prepared from the inorganic polar solvent particularly water is still scarce as regards its* in vivo *and* in vitro* anti-inflammatory activity properties, thus possessing potential research interest.

#### 3.1.4. Immunomodulating Activity

Table 7(d) shows the immunomodulating effect of* C. nutans*. The methanol extract of* C. nutans* has been tested for its immune modulating effect on apoptosis and cytokines expression in experiments on human neutrophils and porcine peripheral blood mononuclear cells (PPBMCs), such as assays for fMLP macrophage activator-induced chemotaxis and chemokinesis, apoptosis, and concanavalin (ConA) and LPS-induced interleukin 10 (IL-10), and tumor necrosis factor-alpha (TNF-*α*) expression [70, 72]. Wanikiat et al. [70] suggested that* C. nutans* methanol extract exhibited dose-dependent suppression of fMLP-induced chemotaxis and chemokinesis of neutrophils without causing the cells to undergo apoptosis.* C. nutans* was found to cause a reduction in IL-10 expression and to have no effect on TNF-*α* expression in PBMC [72]. On the other hand, the ethanolic extract of* C. nutans* has been tested for its immunomodulating effect on cytokines expression in splenocytes, a human keratinocyte cell line (HaCaT), and human peripheral blood mononuclear cells (HPBMC). Tu et al. [52] suggested that low concentrations of the ethanol extract cause IFN-*γ* upregulation while higher concentration of* C. nutans* cause IFN-*γ* downregulation. Sriwanthana et al. [73] studied found that low concentrations of* C. nutans* resulted in increase in lymphocyte proliferation, while higher concentrations of* C. nutans* resulted in decrease of lymphocyte production. The study also found that* C. nutans* did not stimulate an interleukin-2 (IL-2) response or affect the lymphocyte subpopulation, such as total T lymphocytes (CD3), T helper/inducer cells (CD4), T suppressor/cytotoxic cells (CD8), natural killer (NK) cells (CD16/CD56), or B lymphocytes (CD19). On the other hand, 2.5 and 5 mg/mL of ethanol extract increased the interleukin-4 (IL-4) production and 1 and 5 mg/mL of extract suppressed NK activity [73]. Furthermore, 1 and 100 *μ*g/mL* C. nutans* were found to inhibit IFN-*γ* and TNF-*α*-induced keratinocytes apoptosis [63]. A similar trend as in the analgesic and anti-inflammatory properties of* C. nutans* could be observed, where extract prepared from polar solvents exerted better analgesic, anti-inflammatory, and immunomodulating effects. Phenolics, sulfur-containing glucosides, and sulfur-containing compounds might be the main contributors to these activities, as all the mentioned compounds were primarily isolated from polar* C. nutans* extracts. In addition, the described therapeutic effects are always well correlated with the immunology results. Thus, investigations on the biological mechanism of the active constituents present in the polar extract and their effects on immunological cells, mediators, and substances deserve special attention. Exploring the immunological function of other polar extracts, such as water, and nonpolar extracts should be encouraged as well.

#### 3.1.5. Neuromodulating Activity

The efficacy of* C. nutans* in neuro-related protective and modulating functions has also been studied in recent years, as shown in Table 7(e).* In vitro* studies suggested that the neuroprotective effect of* C. nutans* was promising. From the studies by Tan et al. [74] and Tsai et al. [75], the 80% ethanol extract with a dose concentration less than 10 *μ*g/mL was able to suppress post-hypoxic histone deacetylase (HDAC) activation and hypoxic neuronal death in an oxygen-glucose deprivation- (OGD-) reoxygenation assay. It also decreased the levels of cytosolic phospholipase 2 (cPLA2) mRNA expression in mouse primary cortical neurons subjected to 0.5 h of OGD injury. In addition, Tan et al. [74] found that 100 *μ*g/mL 80% ethanol extract was able to suppress histone acetylase (HAT) activity and regulate cPLA2 expression induction in a human neuroblastoma cell line (SH-SY5Y cells) through HDAC inhibitors such as entinostat (MS-275), MC-1568, and trichostatin A (TSA). The research team of Wu et al. [76] further suggested that treating the primary neurons with 0.15–20 *μ*g/mL of 80% ethanol extract 1 h before, at the onset, or after the OGD induced cell death or OGD-reoxygenation treatment was able to mitigate the neuronal apoptosis and protected primary neurons by activating the antiapoptotic activity of peroxisome proliferator-activated receptor-gamma (PPAR-*γ*) → 14-3-3*ε* (antiapoptotic marker), enhancing the C/EBP*β* binding to PPAR-*γ* promoter and amplifying its transcription though the extract was less effective when applied after the OGD treatment. The result also suggested that the protective effect of extract was revoked when cotreated with GW9662 (PPAR-*γ* antagonist). This* in vitro* screening further complemented with the protective effect of extract in attenuating the ischemic brain damage in* in vivo* middle cerebral artery occlusion (MCA) stroke model [76]. Both intracerebroventricular (i.c.v.) infusion (10–60 pg extract) and i.p. injection (24 mg/kg body wt extract) of extract demonstrated that the extract possessed the ability in mitigating apoptotic neuronal death, cerebral infarct volume, and behavioral deficiency in the rat MCA occlusion model. The detailed neuroprotective mechanism of* C. nutans* was similar to* in vitro* OGD study and was elaborated in Table 7(e). In addition, an* in vivo *study conducted by Lau et al. [77] revealed that the three different tested doses of methanol extract were able to stimulate acetylcholinesterase activity in the heart, liver, and kidney, but not the brain of mice without inducing any signs of toxicity in the mice. As a short summary, similar results have been reported as those regarding the analgesic, anti-inflammatory, and immunomodulating activities, where the polar extract of* C. nutans* possessed a great neuroprotective effect. Nevertheless, research on the neuroprotective strength of* C. nutans* is still new and therefore holds enormous potential for different research directions. Further investigation of the potential efficacy of polar* C. nutans* extract in* in vivo* neurology-inflammation related studies might lead to a breakthrough. Research should fill in gaps about the neuro-related function of nonpolar extract as well.

#### 3.1.6. Antidiabetic and *α*-Glucosidase Inhibitory Activity

Table 7(f) shows a list of studies conducted on the antidiabetic and *α*-glucosidase inhibition effects of* C. nutans*. There have been a total of 4* in vitro* studies assessing the hyperglycemia inhibitory activity of* C. nutans *through an *α*-glucosidase inhibition assay. All the results showed that* C. nutans* had a very low inhibitory effect on the *α*-glucosidase enzyme when the employed extracts were prepared using conventional preparation methods [4, 28, 37, 61]. Although IC_50_ was not calculated by Alam et al. [28], a lower IC_50_ value is anticipated as the extract prepared through advanced supercritical extraction exhibited 95.79% of *α*-glucoside inhibition when the stock was prepared at 5000 *μ*g/mL. Despite* C. nutans* not showing particularly pronounced inhibition against *α*-glucosidase, 3* in vivo* antidiabetic experiments suggested that the water extract, 80% methanol extract, and the insoluble ethyl acetate fraction from the 80% ethanol extract were able to attenuate the insulin resistance induced by a high fat and high cholesterol diet (HFHC) and serum glucose level [25, 39]. In general, the high *α*-glucosidase inhibition by SFE treated extracts might indicate that the compounds active against the *α*-glucosidase enzyme are heat-sensitive or easily degradable. Thus, extra precaution is needed during the extract preparation step. On the other hand, conventional method prepared extracts that mimicked the traditional application showed low inhibition towards* in vitro α*-glucosidase tests while traditional uses suggested that* C. nutans* is an antidote for diabetes. The contradictory results might be attributed by improper mode of action of diabetes being investigated. Since *α*-glucosidase inhibition test focuses on mechanism of postprandial hyperglycemia, further study on other mechanism approach, experiment on the reaction of the extract towards *β*-cell in pancreas, test that involved other enzymes in hyperglycemia such as pancreatic *α*-amylase, glucose diffusion assay, glucose uptake by yeast cells, and nonenzymatic glycosylation assay are highly recommended.

#### 3.1.7. Antioxidant Activity

Considerable number of analyses have been done on the antioxidant effect of* C. nutans* as shown in Table 7(g). Based on the 2,2-diphenyl-1-picrylhydrazyl (DPPH) free radical assay, some studies showed that* C. nutans* up to 1 mg/mL is a potential antioxidant agent, while other studies found that the DPPH inhibitory properties of* C. nutans* could not be determined [47, 64, 78]. The data in the table showed that, among the polar and nonpolar extracts tested, the highest antioxidant properties of* C. nutans *was detected in the study by Ghasemzadeh et al. [41], where* C. nutans* bud extract that was cultivated for 1 year at a farm located in Malaysia and that underwent freeze drying and methanol extraction resulted in IC_50_ value of 64.6 *μ*g/mL. The second most common antioxidant determination assay selected by researchers was the ferric reducing antioxidant power (FRAP) assay. However, discussion of the FRAP results is difficult, as there is a lack of unit standardization in the measurement. The results from others* in vitro* antioxidant assays, such as those for hydrogen peroxide scavenging, metal chelating, nitric oxide scavenging, 2,2′-azino-bis(3-ethylbenzothiazoline-6-sulfonic acid) (ABTS) cation radical scavenging, galvinoxyl radical scavenging activity, superoxide radical scavenging activity, and phorbol 12-myristate 13-acetate (PMA) induced peroxide production in rat macrophages and protective effect against peroxyl radicals initiator- (AAPH-) induced oxidative hemolysis, have been summarized in Table 7(g(3)–g(10)). Overall, polar and semipolar solvent-extracted* C. nutans* were more likely to exhibit moderate antioxidant activity at concentration varying from 0.0125 to 10 mg/mL than nonpolar extracts. In addition, there was a recent study that evaluated the antioxidant activity of* C. nutans in vivo* by using a hyperlipidemia-associated oxidative stress model [42]. The study suggested that both the water and 80% methanol leaf extracts at up to 500 mg/kg bw rats were able to mitigate oxidative stress by improving the activity of serum antioxidant enzymes and expression of hepatic antioxidant genes [42]. In summary, polar and semipolar extracts are more likely to exhibit antioxidant properties. A possible hypothesis is that phenolics, sulfur-containing glucosides, sulfur-containing compounds, chlorophyll derivatives, and some phytosterol derivatives that are isolated from* C. nutans* using polar and semipolar solvents might be the contributors. Thus, studies on the antioxidant properties of these isolated compounds and further evaluation of the plant's antioxidant effect in* in vivo* studies are required.

#### 3.1.8. Antiviral Activity

The antimicrobial properties of* C. nutans* have been further classified into antiviral, antibacterial, and antifungal effects, as shown in Tables 7(h), 7(i), and 7(j), respectively. For the antiviral activity of* C. nutans*, the types of virus that have been studied were varicella zoster virus (VZV), herpes simplex virus (HSV), fish pathogenic viruses, crustaceans (shrimp and prawn) infectious viruses, mosquito-borne viruses, and poultry and bird contagious viruses. Notably, the antivirus studies were always accompanied with a prescreening test for cytotoxicity to determine the subtoxic concentration of the test sample and to ensure that the test sample has a killing effect on the virus instead of on the host cell [79]. Researchers have also investigated the mode of action of* C. nutans* in terms of its antiviral potential through 3 different stages of treatment. Pretreatment (pre-) studies are those where the test sample is allowed to incubate with the cell for a time of period before it is infected with the virus. This approach reflects the virucidal activity of the test sample, as the test sample interferes with the viral structure to prevent or inhibit the viral penetrating or adsorbing to the host cell [79]. In posttreatment (post-) studies, instead of being preincubated with the test sample, the virus is attached to the host cell that has first only been cultured with the test sample. The test sample is thus considered to have antiviral activity if it successfully inhibits viral DNA replication [79]. By contrast, for the direct inactivation (direct) pathway, the virus is preincubated with the test samples first before being added to the host cell. The test sample is said to have an inactivation ability against the virus if it interferes with or causes damage to the viral glycoproteins, the virus envelope, or the virus structure before they enter the host cell [79].

Thus far, only one* in vitro* study and a total of 3 clinical trials have been conducted to evaluate* C. nutans'* anti-VZV activity. The* in vitro* result suggested that the organic extract exhibited anti-VZV activity through the direct inactivation stage [80]. In the clinical studies, the* C. nutans* extracts were formulated into a 5%* C. nutans* cream prior to testing its ability to combat VZV infections. The result suggested that the* C. nutans* containing cream was able to exhibit a positive curing effect. All the studies showed that the percentage of patients who experienced lesion crusting within 3 days and lesion healing within 7 days after applying the* C. nutans* cream topically was better than the percentage in the placebo and acyclovir groups. In addition, the pain score and side effect resulting from* C. nutans* cream application were lower [8, 81, 82].

For the anti-HSV-1 activity of* C. nutans*, the ethyl acetate, methanol, dichloromethane, chloroform, and* n*-hexane extracts have been evaluated. Thongchai et al. [79] suggested that the* C. nutans* ethyl acetate leaf extract displayed the best inhibition on HSV strain with IC_50_ value of 7.6 *μ*g/mL through the pretreatment mechanism. Meanwhile, the methanol, ethanol dichloromethane, chloroform, and hexane extracts were also tested for their anti-HSV-2 activity. The* C. nutans* methanol and hexane leaf extracts were found to have better and comparable inhibition against HSV-2-Baylor 186 strain, via posttreatment action, with IC_50_ value of 65.13 and 72.62 *μ*g/mL, respectively [83]. The anti-HSV-2 activity of* C. nutans* has also been evaluated in clinical studies using the 5% extract cream. The results obtained were similar to those from the clinical studies on the anti-VZV activity; when compared to a placebo, the days required for lesion crusting and healing after application of* C. nutans* were higher than or the same in terms of efficacy as acyclovir [82, 84, 85]. Regarding its efficacy in inhibiting fish pathogenic viruses, among the virus strains tested, the* C. nutans *ethanolic extract exerted 100% inhibition on plaque formation in infectious hematopoietic necrosis virus (IHNV) and* Oncorhynchus masou *virus (OMV) strains but not in infectious pancreatic necrosis virus (IPNV) through the direct inactivation stage.

On the other hand, it was also found that* C. nutans* ethanol extract had an excellent protective effect on cultured black tiger shrimp against yellow head rhabdo-like virus (YRV-RNA) through a direct inactivation mechanism and pronounced virucidal activity against dengue virus (IC_50_ value of 31.04 *μ*g/mL); however, it was less effective against Newcastle disease virus (NDV) caused by poultry and birds [52, 86, 87]. In summary,* C. nutans* ethanol extract possessed a very promising antiviral effect. Future research may explicate the antiviral activity of stigmasterol derivative, sulfur-containing compounds, monoacylmonogalactosylgycerol, and all of nine cerebrosides, as, according to previous studies, they are the main compounds isolated from the ethanol extract. Though clinical trial suggested its therapeutic properties, research that optimizes the efficacy dosage and profiles the safety uses of the plant extract is lacking.

#### 3.1.9. Anti-Bacterial Activity

Table 7(i) shows the antibacterial properties of* C. nutans*. Overall, studies were mainly based on* in vitro* assays and only polar (water, methanol, and ethanol) and semipolar (chloroform and ethyl acetate) extracts, but nonpolar* C. nutans* extracts not were evaluated for their anti-bacterial activity. A total of 9 different Gram-positive bacteria have been used in previous studies, including* Bacillus cereus, Bacillus subtilis, Staphylococcus aureus,* methicillin-resistant* Staphylococcus aureus* (MRSA), methicillin-sensitive* Staphylococcus aureus* (MSSA),* Micrococcus luteus, Propionibacterium acnes, Staphylococcus epidermidis*, and* Streptococcus* sp. On the other hand,* Aeromonas hydrophila, Escherichia coli* and its strains,* Neisseria gonorrhoeae*,* Pseudomonas aeruginosa* and its strains,* Salmonella enterica* and its serovars, and* Vibrio harveyi *and* Vibrio parahaemolyticus* were the Gram-positive bacteria selected for study. As a summary of the antibacterial efficacy of* C. nutans*, the most effective extract of the plant regarding antibacterial effect was prepared by oven-drying and undergoing ethyl acetate fractionation. The extract prevented visible* B. cereus, E. coli, *and* S. enterica Typhimurium* growth at 1.39 mg/mL [88].

#### 3.1.10. Antifungal Activity

Concerning the antifungal efficacy of* C. nutans, *limited studies had been carried out to date. In the two reported antifungal activity studies, Cheeptham and Towers [89] found that* C. nutans* did not exert a fungicidal effect on* Candida albicans* or* Aspergillus fumigatus *when 5 mg/mL 95% ethanol leaf extract was tested. By contrast, a fraction of the ethyl acetate extract at the minimal concentration of 1.39 mg/mL did exhibit an antifungal effect on* C. albicans *[88]. In summary, based on the available* in vitro* studies,* C. nutans* polar and semipolar extracts exhibited a weak antibacterial and antifungal activities. Any further research should focus on assessing the biological action of the polar and semipolar extracts of* C. nutans* in inhibiting bacterial and fungal infections as well as augmenting the limited information on the antibacterial and antifungal potency of the nonpolar extract.

#### 3.1.11. Anticancer Activity

A total of 15 types of cancer cells that are responsible for different types of cancer have been used to evaluate the* in vitro* anticancer effect of* C. nutans*, as summarized in Table 7(k). These include human melanoma cell lines (D24 and MM418C1) for skin cancer, a human osteosarcoma cell line (cultured Saos-2) for bone tumor, human breast adenocarcinoma (MCF7) and human breast carcinoma (BT474) cell line for breast cancer, human lung adenocarcinoma (NCI-H23) and human lung carcinoma (NCI-H460) cell lines for lung cancer, a human liver hepatocellular carcinoma cell line (HepG2) for liver cancer, a human neuroblastoma cell line (IMR-32) for nerve tissue cancer, a human gastric cancer cell line (SNU-1) for gastric cancer, human colon adenocarcinoma (LS-174T) and human colorectal carcinoma cell line (HCT 116) for colon and rectal cancer, a human cervical cancer cell line (HeLa) for cervical cancer, a human erythroleukemia cell line (K562) for acute myeloid leukemia, and a human Burkitt's lymphoma cell line (Raji) for lymphatic disorder. In addition, there were 2 studies that assessed the* in vivo* antimutagenic and anticancer effect of* C. nutans* [33, 90]. Based on these studies, the most effective extract was the petroleum ether leaf extract, which showed the strongest cytotoxic activity after 72 h of incubation. The concentration of the extract that caused 50% death (CC_50_) was 18.0 *μ*g/mL in HeLa cell and 20.0 *μ*g/mL in the K-562 cell line [88]. According to the National Cancer Institute (NCI), a crude extract that exhibits an IC_50_ value <20 *μ*g/mL can be considered as an active anticancer agent [41]. Overall, nonpolar leaf extract possessed a very pronounced cytotoxic effect on cervical and erythroleukemia cancerous cell. Hence, to evaluate the suitability of* C. nutans* to involve in anticancer drug development, further* in vivo* clinical experimental studies on cervical cancer and erythroleukemia models are encouraged. The mechanism behinds the activity should be critically analyzed too. On the other hand, from the summarized data, observed inconsistency of incubation period during the experiment might be one of the factors that caused result deviation. Further parameter standardization should be implemented. Furthermore, the effect of the ethanol extract on* in vivo* hepatocarcinoma tumor-bearing mice exhibited a similar trend as in the* in vitro* study (methanolic extract) suggesting that both polar extracts possessed similar cytotoxic action towards liver cancerous cells, which is worthy of further investigation.

#### 3.1.12. Wound Healing Activity

In addition to the other well studied pharmacological activities of* C. nutans*, there are also a few other potential pharmacological activities which are less studied or that even have been reported only once. Table 7(l) summarizes the wound healing ability of* C. nutans. *Between the chloroform extract and hexane extract, the chloroform extract (10 *μ*g/mL) was found to provide the best improvement in human gingival fibroblast (HGF) migration rate and wound recovery in the 6 h of observation [11]. Before labelling it as a potential wound healing agent during inflammation, however, more studies are needed to verify its wound healing effect and determine which of the chlorophyll derivatives, diglycerides, or stigmasterol derivatives are the main factor for this positive activity.

#### 3.1.13. Protective Effect on Plasmid DNA Activity

The protective effect of* C. nutans *on plasmid DNA is tabulated in Table 7(m). Yuann et al. [64] suggested that, compared to green tea extract (protection up to 30 min), the 70% ethanol extract had the ability to reduce the number of DNA cleavages, preserve higher levels of supercoiled plasmid DNA integrity, and provide better protection against the riboflavin photoreaction induced superoxide for up to 50 min. In short, the polar extract showed a protective effect on plasmid DNA, but more analysis involving an* in vivo* model is needed. In addition, it is important to direct research focus towards the protective effect of the nonpolar extract as well.

#### 3.1.14. Lipid Elevated Inhibition Activity

The ability of* C. nutans* to attenuate obesity had been assessed as shown in Table 7(n) through an* in vitro* porcine pancreatic lipase inhibition assay. The result suggested that, instead of an inhibitory effect, the methanol extract exhibited a pancreatic lipase promoting effect [21]. However, as suggested previously, more research involving cellular and* in vivo *experimental design are encouraged before reaching any conclusion.

#### 3.1.15. Oral Mucositis and Stomatitis Treatment

*C. nutans *is also formulated for oral application to treat radiation induced oral mucositis in head and neck cancer patients and in patients suffering from recurrent aphthous stomatitis, as presented in Table 7(o) [10, 91]. The results revealed that, compared to the positive control group, the time onset of oral mucositis was significantly later, and the pain score was significantly lower [10]. Although it is not as efficacious as the positive control group (triamcinolone acetonide), it can be a good alternative to lessen the severity of ulcers [91].

### 3.2. Pharmacological Activity of* C. nutans* Isolated Pure Compounds

After understanding the pharmacological properties of* C. nutans* extract, it is beneficial to further investigate which compound in a particular extract is responsible for the therapeutic activity of the plant.  Table 8 summarizes the pharmacological activities of the isolated compounds from* C. nutan*s. For anti-inflammatory activity, only cerebrosides from the ethanol extract have been studied, and they were reported as being ineffective in suppressing cyclooxygenase (COX-1 and COX-2) induced prostaglandin E2 generation and HSV-1 infection [55]. The result obtained was inconsistent with the anti-inflammatory effect of the extract observed previously. Nevertheless, the type of experiment conducted might cause this variation, and further attention should focused on the anti-inflammatory effect of other compounds isolated from the polar solvent. Le et al. [45] studied the immunomodulating function of flavones (schaftoside) and terpenes (stigmasterol, *β*-sitosterol, and lupeol) compounds isolated from the hexane fraction of the methanol extract. The results showed that only stigmasterol and *β*-sitosterol were able to suppress the T cell proliferation mediated by ConA and that only *β*-sitosterol exerted an immune suppressive effect on T helper 2 (Th2) cytokines (IL-4 and IL-10). A similar trend was observed with the polar extract of* C. nutans* which exerted a better immunomodulating effect than the nonpolar extract. Clinamide D, which was isolated from the methanol extract, was found to have a moderate antioxidant effect with 76.05 ± 0.02% DPPH radical inhibition at 1000 *μ*g/mL which is consistent with the good DPPH inhibition properties of the methanol extract [41, 53].

Regarding the antiviral activity, 13^2^-hydroxy-(13^2^-R)-phaeophytin b from the chloroform extract was found to possess a better anti-HSV-1 effect with an IC_50_ value of 1.96 nM through direct inactivation [107]. Pongmuangmul et al. [56] showed that monogalactosyl diglyceride (MGDG) and digalactosyl diglyceride (DGDG) isolated from the chloroform extract exhibited promising anti HSV-1 and anti-HSV-2 properties in the poststep of infection, where the IC_50_ values were in a range from 36.00 *μ*g/mL to 43.20 *μ*g/mL. Sittiso et al. [108] suggested that phaeophorbide A from the chloroform extract was able to inhibit dengue viral 2 replication in the direct inactivation and postincubation stages with a CC_50_ of 25 *μ*g/mL. Based on this study, it can be observed that, in antiviral studies, compounds isolated from the chloroform extract are more likely to exhibit antiviral activity whereas the ethyl acetate, methanol, and ethanol extracts were more likely to possess anti-HSV-1 and anti-HSV-2 activities, and the ethanol extract was more likely to possess an anti-dengue inhibitory effect. Thus, it might be a valuable approach to investigate the relationship between that antiviral properties of compounds isolated from the aforementioned extracts. Tinh [49] found that 3-amino-4,5-dihydroxyfuran-2(3H)-one isolated from the ethanol extract exhibited moderate antibacterial activity against* S. aureus *(MIC equal to 0.3125 mg/mL). This result was inconsistent with the antibacterial activity of the corresponding extract against the same bacterial strain. This might due to the antibacterial properties of the crude extract being hindered by the other constituents present in the extract. In addition, phaeophorbide A from the chloroform extract was found to possess a promising* in vitro *anticancer effect on human lung carcinoma cell (A549 cells) at a CC_50_ equal to 25 *μ*g/mL [108]. This result was consistent with the study from Yong et al. [32] which found that the chloroform extract at 100 *μ*g/mL showed 55.82% inhibition of human lung cancer cell. A novel polysaccharide-peptide complex (CNP-1-2) isolated by Huang et al. [59] from the 70% ethanol extract also exhibited potential anticancer activity on a human gastric cancer cell line (SGC-7901 cells), where, at concentration of 200 *μ*g/mL, it inhibited cell growth by 92.34 ± 0.94% after 48 h of incubation. By contrast, Yong et al. [32] found that the chloroform extract exhibited a weak killing effect on human gastric cancer cells (31.25 ± 1.09% at 100 *μ*g/mL).

## 4. Conclusion

Phytochemical identification and quantification have suggested that* C. nutans* is a rich source of phenolics, flavonoids, triterpenoids, and chlorophyll derivatives; however, their retention in an extract is largely influenced by the plant preparation methods. On the other hand, pharmacological studies on* C. nutans* have suggested that its polar extracts can be a promising anti-inflammation, antiviral, anticancer, immune- and neuromodulating, and plasmid DNA protective agents, as well as a moderate antivenom, analgesic, antidiabetic, and antioxidant agent and a weak lipid elevating inhibitor. By contrast, its semipolar extracts can be a promising antiviral, anticancer and wound healing agent, a moderate anti-inflammation and antioxidant agent, and a weak antibacterial and antifungal agent, and its nonpolar extracts can be a strong anticancer agent. However, as insufficient previous scientific studies have been conducted and most experiments were preliminary and fundamentally oriented, more sophisticated evaluation and pathway analyses of the aforementioned biological and therapeutic potential of this plant are urged before implementing it in the pharmaceutical and cosmetics industries. In addition, since* in vitro* assays do not fully mimic the physiological environment in animals and humans, additional cellular,* in vivo *and clinical trials are likewise to fully interpret the effect of* C. nutans* on disease inhibition and prevention. Mechanism of action of the extract towards particular treatment should explicate. It is also likely to have more experimental studies that could substantiate and describe the correlation of the isolated phytochemicals from* C. nutans *with their corresponding pharmacological effects. A note for future researchers: it is of utmost importance to provide complete data such as the extract concentration, the extraction solvent used, and the experimental design, which was found to be lacking throughout the data searching. All the aforementioned data could be a crucial point that determines the accuracy of the interpretation of the results, the credibility of the study, and the reproducibility of the work in the future. In conclusion, through this review, the authors hope to provide a more systematic summary of the previous* C. nutans* works according to the experimental design and plant preparation methods that have been thus far done in phytochemical and pharmacological relevant* C. nutans* studies. The authors also anticipate to provide some possible idea for researchers regarding the future research perception and direction of this plant.

## Figures and Tables

**Table 1:** Phytochemical screening test of *C. nutans.*

Chemical class	Origin	Extract	Present/Absent
Alkaloid	Indonesia^1^	Leaf-water extract^1^	Present^1^
	Malaysia^2,3,5^	Leaf-100% methanol extract^2,5^	Absent^2,5^
		Leaf-100% chloroform extract^3^	Presence^3^
		Leaf-70% methanol extract^4^	Absent^4^
Saponin	Indonesia^1^	Leaf-water extract^1^	Absent^1^
	Malaysia^2,3,4,5^	Leaf-100% methanol extract^2, 5^	Present^2,5^
		Leaf-100% chloroform extract^3^	Absent^3^
		Leaf-70% methanol extract^4^	Absent^4^
Flavonoids	Indonesia^1^	Leaf-water extract^1^	Present^1^
	Malaysia^2,3,4,5^	Leaf-100% methanol extract^2,5^	Present^2,5^
		Leaf-100% chloroform extract^3^	Present^4^
		Leaf-70% methanol extract^4^	Present^3^
Triterpenoids	Indonesia^1^	Leaf-water extract^1^	Present^1^
	Malaysia^5^	Leaf-100% methanol extract^5^	Present^2^
Diterpenes	Malaysia^2^	Leaf-100% methanol extract^2^	Present
Steroids	Indonesia^1^	Leaf-water extract^1^	Present^1^
	Malaysia^4,5^	Leaf-70% methanol extract^4^	Present^2,3^
	Thailand^7^	Leaf-100% methanol extract^5^	Present^5^
		Stem-Light petroleum extract^7^	Present^7^
Phytosterol	Malaysia^2,4^	Leaf-100% methanol extract^2^	Present^1^
		Leaf-70% methanol extract^4^	Present^2^
Tannin	Indonesia^1^	Leaf-water extract^1^	Present^1^
	Malaysia^2,3,4,5^	Leaf-100% methanol extract^2,5^	Absent^2,5^
		Leaf-100% chloroform extract^3^	Present^3^
		Leaf-70% methanol extract^4^	Present^4^
Quinone	Indonesia^1^	Leaf-water extract^1^	Absent
Phenolic compound	Malaysia^2^	Leaf-100% methanol extract^2^	Present
Glycosides	Malaysia^3,4^	Leaf-100% chloroform extract^3^	Present^1^
		Leaf-70% methanol extract^4^	Present^2^
Iridoids	Thailand^6^	Leaf ^6^	Present^6^
Carbohydrates	Malaysia^4^	Leaf-70% methanol extract^4^	Present^4^
Protein and amino acids	Malaysia^4^	Leaf-70% methanol extract^4^	Present^4^

*Reference*. ^1^Nurulita et al. [25]; ^2^Yang et al. [6]; ^3^Goonasakaran [26]; ^4^Sekar and Rashid [22]; ^5^Abdul Rahim et al. [19]; ^6^Keawpradub and Purintrapiban [20]; ^7^Dampawan et al. [27].

**Table:** (a) Phytochemical identification based on column chromatography isolation and spectroscopy structural elucidation

Phytochemical	Plant part	Postharvesting method	Extract/Fraction	Plant origin	Reference
Terpenes-Triterpenoids					

Lupeol	Stem^2^	Air Dry; Soxhlet^2^	Petroleum ether extract^1^	Thailand^1,2^	[44]^1^
	Leaf^3^	Oven Dry; Soaking^3^	Light petroleum extract^2^	Seremban Malaysia^3^	[27]^2^
			Hexane fraction of methanol extract^3^		[45]^3^
Betulin	Root	-	-	China	[46]

Terpenes-Phytosterols					

*β*-sitosterol	Stem^2^	Air Dry; Soxhlet^2^	Petroleum ether extract^1^	Thailand^1,2^	[44]^1^
	Leaf^3^	Oven Dry; Soaking^3^	Light petroleum extract^2^	Seremban Malaysia^3^	[27]^2^
			Hexane fraction of methanol extract^3^		[45]^3^
*β*-Sitosterol-3-O-*β* glucopyranoside	Stem	Sun Dry; Soaking	Sub-fraction of methanol extract	Vietnam	[47]
*β*-Sitosterol-3-O-*β* glucoside	Stem	Sun Dry; Soaking	Sub-fraction of methanol extract	Vietnam	[47]
Stigmasterol	Stem^2^	Oven Dry; Soaking^3^	Petroleum ether extract^1^	Thailand^1,2^	[44]^1^
	Leaf^3^		Light petroleum extract^2^	Malaysia^3^	[48]^2^
			Hexane fraction of methanol extract^3^		[45]^3^
Stigmasterol*-β-D-*glucoside	Aerial	Sun Dry; Maceration	Methanol fraction of 96% ethanol extract	Vietnam	[49]
Stigmasteryl-3-*O*-*β*-*D*-glucopyranoside	Leaf	Oven Dry; Soxhlet	Chloroform extract	Thailand	[9]

Phenolics compounds					

Shaftoside^1,2,3^, vitexin^1,2^, isovitexin^1,2^, isomollupentin 7-*O-β*-glucopyranoside^1^, orientin^1,2^, isoorientin^1,2^, gallic acid^2^, apigenin 6,8-di-*C*-*α*-L-arabinopyranoside^2^	Aerial^1,2^	Reflux^2^	Butanol soluble portion of methanol	Thailand^1^	[50]^1^
		Oven Dry; Soaking^3^	extract^1^	Seremban Malaysia^2,3^	[33]^2^
	Leaf^3^	-	30% ethanol extract^2^		[45]^3^
			Hexane fraction of methanol extract^3^		

Sulfur-containing glucosides compounds					

Clinacoside A, Clinacoside B, Clinacoside C, Cycloclinacoside A1, Cycloclinacoside A2	Aerial	-	Butanol soluble and aqueous soluble portion of methanol extract	Thailand	[51]

Sulfur-containing compounds					

Clinamides A^1^, Clinamides B^1^, Clinamides C^1^, Clinamides D^2^, Clinamides E^2^, 2-*cis*-entadamide A^1^, Entadamide A^1^, Entadamide C^1^	Aerial^1^	Air Dry; Soaking^1^	80% Ethanol extract^1^	Taiwan^1^	[52]^1^
	Whole plant^2^	Sonication^2^	Methanol extract^2^	Jelebu, Malaysia^2^	[53]^2^

Lipids					

Myricyl alcohol	-	-	Petroleum ether extract	Thailand	[44]
1,2-*O*-dilinolenoyl-3-*O-β-D*-galactopyranosyl-glycerol, 1-*O*-palmitoyl-2-*O*-linolenoyl-3-*O*-(*α*-D-galactopyranosyl-(1′′→6′)-*O-β-D*-galacctopyranosyl)-glycerol	Leaf	-	-	Thailand	[54]
Monoacylmonogalactosylglycerol, a mixture of nine cerebrosides	Leaf	-	Ethyl acetate-soluble fraction of 95% ethanol extract	Thailand	[55]
Monogalactosyl diglyceride, digalactosyl diglyceride	Leaf	Soxhlet extraction	Chloroform extract	Thailand	[56]

Chlorophyll a and b derivatives					

Purpurin 18 phytyl ester^1,2,4^ Phaeophorbide a^1,2,4^ 13^2^-hydroxy-(13^2^-*S*)-chlorophyll b^3,4^ 13^2^-hydroxy-(13^2^-*R*)-chlorophyll b^3,4^ 13^2^-hydroxy-(13^2^-S)-phaeophytin b^1,2,4^ 13^2^-hydroxy-(13^2^-*R*)-phaeophytin b^3,4^ 13^2^-hydroxy-(13^2^-*S*)-phaeophytin a^3,4^ 13^2^-hydroxy-(13^2^-*R*)-phaeophytin a^3,4^	Leaf^1,2,3,4^	Oven Dry; Soxhlet^1,2,3,4^	Chloroform extract^1,2,3,4^	Thailand^1,2,3,4^	[57]^1^ [17]^2^ [9]^3^ [58]^4^

Others					

*trans*-3-methylsulfinyl-2-propenol	Aerial	Air Dry; Soaking	80% Ethanol extract	Taiwan	[52]
3-amino-4,5-dihydroxyfuran-2(3H)-one	Aerial	Sun Dry; Maceration	Methanol fraction of 96% ethanol extract	Vietnam	[49]
polysaccharide–peptide complex	Leaf	Oven Dry	Hot water and 75% ethanol precipitation extract	Seremban Malaysia	[59]

**Table:** (b) Phytochemical identification based on spectrometry and spectroscopy identification

Phyto-constituents	Plant part	Post-harvesting method	Extract	Plant origin	Analytical tools	Reference
2-ethyl-oxetane, 9,12,15-octadecatrienoic acid, 2,3-dimethylpyridine, 3-deoxyd-mannoic lactone, neophytadiene, phytol, 2,3-dihydrobenzofuran, *n*-hexadecanoic acid	Leaf	Oven dry; Soaking	Abs methanol	Kuala Lumpur Malaysia	GCMS	[19]
*n*-Pentadecanol, eicosane, 1-nonadecene, heptadecane, dibutylphthalate, *n*-Tetracosanol-1, heneicosane, behenic alcohol, 1-heptacosanol, 1,2-Benzenedicarboxylic acid, mono (2-ethylhexyl) ester, nonadecyl heptafluorobutyrate, eicosayl trifluoroacetate, 1,2-benzenedicarcoxylic acid, dinonyl ester, phthalic acid, dodecyl nonylester	Leaf	Oven dry	Chloroform	Selangor Malaysia	GCMS	[32]
Squalene, *β*-tocopherol, vitamin E, Campsterol, stigmasterol, *γ*-sitosterol, *β*-amyrin, *α*-amyrin, lupeol, propanoic acid, betulin	Leaf, stem	Combination of air dry and freeze dry; Maceration	Abs methanol, abs ethyl acetate	Sabah Malaysia	GCMS	[29]
Lactic Acid, Glycolic acid, 3-Pyridinol, Glycerol, Phosphonic acid, Catechol, Butanedioic acid, Glyceric acid, Erythrono-1,4-lactone, (Z), Malic acid, Benzaldehyde, 3-methoxy-4-[(trimethylsilyl)oxy]-, O-methyloxime, Cyclooctasiloxane, Levoglucosan, Vanillic Acid, D-Ribo-Hexonic acid, 3-deoxy-2,5,6-tris-O-(trimethylsilyl)-, lactone, D-(-)-Fructofuranose, pentakis(trimethylsilyl) ether (isomer 1), Myristic acid, D-Pinitol, Neophytadiene, D-Fructose, 1,3,4,5,6-pentakis-O-(trimethylsilyl)-, O-methyloxime, Syringic acid, D-Fructose, 1,3,4,5,6-pentakis-O-(trimethylsilyl)-, O-methyloxime, d-Glucose, 2,3,4,5,6-pentakis-O-(trimethylsilyl)-, o-methyloxyme, (1Z)-, Gallic acid, Muco-Inositol, D-Glucose, Heptadecanoic acid, Phytol, 9,12-Octadecadienoic acid (Z,Z)-, Oleic Acid, L-Rhamnose, Cyclononasiloxane, octadecamethyl-, Octadecane-1,2-diol, Ricinoleic acid, Arachidic acid, L-Rhamnose, 1,2-Benzenedicarboxylic acid, mono(1-methylheptyl) ester, (+/−),1-Monopalmitin, Cyclononasiloxane, octadecamethyl-, 1-Monolinolein, Glycerol monostearate, Squalene, *β*-Gentiobiose, B-tocopherol, *γ*-tocopherol, *α*-Tocopherol, Campesterol	Stem	Air dry; Supercritical-soxhlet extraction	-	Seremban Malaysia	GC-QTOF-MS	[28]
Vitamin E, campesterol, stigmasterol, *γ*-Sitosterol, *β*-Sitosterol, *β*-amyrin, lupeol, betulin	Root	Freeze dry; Soaking	Methanol	Sabah Malaysia	GCMS	[31]
Oleic acid, squalene, vitamin E, campesterol, stigmasterol, *γ*-stosterol, *β*-sitosterol, lup-20(29)-en-3-one, *β*-amyrin, lupeol, lup-20(29)-en-3-ol-acetate, betulin	Root	Freeze dry; Soaking	Ethyl acetate	Sabah Malaysia	GCMS	[31]
Neophytadiene, iron, 7,9-Dodecadien-1-ol, Myristic acid, Palmitic acid, Palmitic acid-methyl ester, Benzenethanol, Phytol, Squalene, Stearic acid-methyl ester, Margaric acid-ethyl ester, Lupeol, Linoleic acid-ethyl ester, Linolenic acid-methyl ester, 2-Butanol, Butanamide, 2-cyclopenten-1-one-2-hydroxy, Glycine, Pentanal, Isoveraldehyde, Dimethyl trisulfide, Thiophene, Succinic acid, Glycolic acid, Oxazolidine, Thiophene, 9-Azabicyclo (6.1.0) non-4-4en-9-amine, 4-Vinyl-2-methoxy-phenol, Phenol,2,6-dimethoxy, 4H-Pyran-4-one,2,3-dihydro-3,5-dihydroxy-6-methyl, Glycerine, 4-Vinylphenol	Aerial	Oven dry; MAE, SFE, Soxhlet	Aqueous ethanol	Kuala Lumpur Malaysia	GCMS	[30]
Isoorientin, orientin, isovitexin, vitexin, schaftoside, 6, 8-apigenin-*C-α-L*-pyranarabinoside	Aerial	-	30% Ethanol	Seremban Malaysia	HPLC LCMS/MS	[33]
Kaempferol-7-neohesperidoside, Isoschaftoside, Isoorientin, Vitexin, 3′,7-Dimethoxy-3-hydroxyflavone, 2′,6-Dihydroxyflavone, (+)−Catechin, 7-Hydroxyflavone, Gallic acid, Flavanone-7-*O*-glycoside	Aerial	Oven dry; MAE, SFE, Soxhlet	86% Ethanol, 50% Ethanol	KL Malaysia	UPLC-ESI-QTOF/MS	[30]
Schaftoside, Arabinoysl–glucosyl apigenin isomer, Ascorbic acid, Gendarucin A, Gendarucin A isomer, 3, 3-di-*O*-methylellagic acid, Methyl 2-[cyclohex-2-en-1-yl(hydroxyl) methyl]-3-hydroxy-4-(2-hydroxyethyl)-3-methyl-5-oxoprolinate, Methyl 2-[cyclohex-2-en-1-yl(hydroxyl) methyl]-3-hydroxy-4-(2-hydroxyethyl)-3-methyl-5-oxoprolinate isomer	Leaf	Air dry; Sonication	70% Ethanol	Seremban Malaysia	UPLC-MS/MS	[4]
Gallic acid, 4-hydroxybenzoic acid, caffeic acid, coumaric acid, ferulic acid, schaftoside, vitexin, orientin, isoorientin, isovitexin, luteolin, apigenin, forsythosides H, forsythosides I, diosmetin glycoside, diosmetin.	Leaf	Oven dry; Soaking	100% Methanol	Kuala Lumpur Malaysia	UHPLC-MS	[19]
*Primary metabolites*: Fructose, *α*-Glucose, *β*-Glucose, Sucrose, a mixture of cerebrosides, Monoacylmonogalactosylglycerol, Alanine, Glutamate, Glutamine, Proline, Threonine, Tryptophan, Valine, Citric acid, Formic acid, Fumaric acid, Choline, Adenine, Fatty acid, Ascorbic acid *Secondary metabolites*: Betulin, Lupeol, Stigmasterol, *β*-sitosterol, Clinacoside A, Clinacoside B, Clinacoside C, Cycloclinacoside A1, Cycloclinacoside A2, Isoorientin, Isovitexin, Orientin, Vitexin, Schaftoside, Gendarucin A, Catechin, Quercetin, Quercetin 3-*O*-rhamnoside, Quercetin 3-*O*-arabinofuranoside, Rutin, Chlorogenic acid, Gallic acid	Leaf, Stem	Air dry, Oven dry, Freeze dry; Sonication, Soaking	70% Ethanol	Seremban Malaysia	^1^H-NMR	[4]

1, 2, 3, 4: in each phytochemical class, every superscript number attached to a particular phytochemical, plant part, postharvesting method, extract/ fraction, or plant origin indicated that such information is extracted from the reference which has attached with the same superscript number.

**Table 3:** Total phenolic content (TPC) of *C. nutans.*

Extract	Plant part	Pre- or post-harvesting method	Plant origin	Results of TPC	Extract with highest TPC	Extract with lowest TPC	Reference
Hot water	-	Oven dry; Heating at 90°C	Malaysia	14.70 ± 0.08 mg GAE/g DM	-	-	[37]
Hot water	Leaf	Unfermented and fermented; Microwave-oven dry, freeze dry; Hot boiling water (100°C); Infusion for 1, 2, 5, 10, 15, and 20 min	Sabah Malaysia	88.56 ± 4.40 to 177.80 ± 19.10 mg TAE/L	Unfermented-microwave oven-20 min infusion	Fermented-freeze dry-1 min infusion	[36]
Distilled water	Leaf	Combination of air dry and oven dry; Maceration with shaker-0.5, 1, 3, 5, 24 h	Thailand	26.53 ± 8.83 to 46.71 ± 9.31 mg GAE/g DW	Maceration for 1 h	Maceration for 5 h	[60]
Hot water (70°C), water, methanol, 80% methanol, ethyl acetate, hexane	Leaf, stem	Freeze dry; Sonication	Seremban Malaysia	23.15 ± 2.78 to 73.33 ± 12.18 mg GAE/g extract	80% methanol leaf	Hexane stem	[42]
Hot distilled water, cold distilled water, methanol, ethanol, dichloromethane	Leaf	Orbital shaker	Seremban Malaysia	24.57 ± 0.07 to 48.08 ± 0.04 mg GAE/g DW extract	Cold distilled water	Ethanol	[18]
100% Methanol	Leaf	Maceration	Seremban Malaysia	1.77 ± 0.01 mg GAE/g extract	-	-	[6]
100% Methanol	Leaf	Air dry; Maceration	Seremban Malaysia	0.78 ± 0.01 mg GAE/g DW extract at stock: 10 mg/mL	-	-	[24]
Methanol	Leaf, stem	Oven dry; Sonication	Seremban Malaysia	0.12 and 2.68 mg GAE/g DW sample	Leaf	Stem	[61]
Methanol	Leaf	Air dry; Orbital shaker	Malaysia, Thailand, Vietnam	8.29 to 72.16 mg GAE/g DW extract	Chiang Dao, Thailand	Map Khae, Thialand	[18]
Methanol	Leaf	Oven dry 40°C, 50°C, 60°C, 70°C, 80°C, 100°C; Orbital shaker	Seremban Malaysia	22.44 ± 0.03 to 63.31 ± 0.03 mg GAE/g DW extract	Oven dry at 100°C	Oven dry at 40°C	[18]
Methanol	Leaf, bud	1, 6 and 12 months old; Freeze dry; Shaking	Serdang Malaysia	6.840 ± 0.470 to 15.460 ± 1.231 mg/g DW	6-month-old buds	1-month-old buds	[41]
Polar (methanol and dichloromethane), non-polar (hexane and diethyl ether)	Leaf, stem	Soaking	Pahang Malaysia	1.43 ± 0.1 to 7.99 ± 0.6 mg GAE/g DM	Leaf-polar	Stem-non polar	[62]
Methanol, ethyl acetate, chloroform, hexane fraction	-	Sonication	Malaysia	22.17 ± 0.02 to 119.29 ± 0.07 mg GAE/g DW	Chloroform	Ethyl acetate	[35]
100% Ethanol	Leaf	Maceration	Thailand	4.67 ± 3.60 mg GAE/g wet weight sample	-	-	[63]
100%, 86%, 65%, 50%, 44% Ethanol	Aerial	Oven dry; MAE, p-MAE, SFE, soxhlet extraction	KL Malaysia	5.74 ± 0.29 to 14.56 ± 0.77 mg GAE/g DM	p-MAE in 50% ethanol	MAE in 86% ethanol	[30]
70% Ethanol	Leaf	Freeze dry; Sonication	Perak Malaysia	23.5 mg GAE/g DM	-	-	[64]
70% Ethanol	Leaf, Stem	Freeze dry, oven dry, air dry; Sonication, soaking	Seremban Malaysia	1.04 ± 0.02 to 7.29 ± 0.11 mg GAE/g DW sample	Leaf-Air dry-Sonication	Stem-Freeze dry-Soaking	[4]
-	Stem	Air dry; CO_2_-Soxhlet, Maceration	Seremban Malaysia	CO_2_-Soxhlet: 49.45 mg GAE/g DM, Maceration: 7.54 mg GAE/g DM	-	-	[28]
80% Ethanol	Leaf, stem	Young and mature; Stored for 1, 2, 3, and 4 days	Malaysia	*Maturity:* 60.75 to 117.00 mg GAE/100 g fresh sample *Storage duration:* 71.08 to 140.92 mg GAE/100 g fresh sample	*Maturity* young leaf *Storage* 1 day storage	*Maturity* matured stem *Storage* 4 days storage	[38]

KL: Kuala Lumpur.

**Table 4:** Total flavonoid content (TFC) of *C. nutans*.

Extract	Plant part	Pre- or post- harvesting method	Plant origin	Results of TFC	Extract with highest TFC	Extract with lowest TFC	Reference
Hot water	-	Oven dry; Heating at 90°C	Malaysia	2.07 ± 0.05 mg QE/g dry material	-	-	[37]
Hot water	Leaf	Unfermented and fermented; Microwave-oven dry, freeze dry; hot boiling water (100°C); Infusion for 1, 2, 5, 10, 15, and 20 min	Sabah Malaysia	14.57 ± 0.42 to 22.13 ± 1.53 mg CE/L	Fermented leaf-microwave-oven dry-10 min infusion	Unfermented leaf-microwave oven dry-1 min infusion	[36]
Hot distilled water, cold distilled water, methanol, ethanol, dichloromethane	Leaf	Orbital shaker	Seremban Malaysia	7.09 ± 2.98 to 14.66 ± 1.71 mg QE/g of dry extract	Cold distilled water	Ethanol extract	[18]
100% Methanol	Leaf	Maceration	Seremban Malaysia	0.04 ± 0.001 mg QE/g extract	-	-	[6]
100% Methanol	Leaf	Air dry; Maceration	Seremban Malaysia	0.21 ± 0.005 mg QE/g extract	-	-	[24]
Methanol	Leaf	Air dry; orbital shaker	Malaysia, Thailand, Vietnam	2.96 ± 0.03 to 58.38 ± 0.19 mg QE/g of dry extract	Chiang Dao, Thailand	Map Khae, Thailand	[18]
Methanol	Leaf	Oven dry 40°C, 50°C, 60°C, 70°C, 80°C, 100°C; orbital shaker	Seremban Malaysia	14.02 ± 1.68 to 27.72 ± 0.14 mg QE/g of dry extract	Oven dry at 80°C	Oven dry at 40°C	[18]
Methanol	Leaf, bud	1, 6 and 12 months old; Freeze dry; Shaking	Serdang Malaysia	3.27 ± 0.33 to 6.32 ± 0.74 mg QE/g dry weight	6-month-old buds	1-month-old buds	[41]
Polar (methanol and dichloromethane), non-polar (hexane and diethyl ether)	Leaf, stem	Soaking	Pahang Malaysia	3.27 ± 1.10 to 16.09 ± 4.20 mg QE/g DM	Polar leaf	Non-polar stem	[62]
Methanol, ethyl acetate, chloroform, hexane fraction	-	Sonication	Malaysia	428.67 ± 0.03 to 937.67 ± 0.02 mg BHTE/g	Chloroform fraction	Methanol fraction	[35]
100%, 86%, 65%, 50%, 44% Ethanol	Aerial	Oven dry; MAE, p-MAE, SFE, Soxhlet extraction	KL Malaysia	2.71 ± 0.47 to 5.88 ± 0.22 mg QE/g of dried material	SFE	p-MAE at 86% ethanol	[30]
80% Ethanol	Leaf, stem	Young and mature; Stored for 1, 2, 3, and 4 days	Malaysia	*Maturity* 45.56 to 99.47 mg RE/100 g fresh sample *Storage duration* 41.48 to 116.36 mg RE/100 g fresh sample	*Maturity* young leaf *Storage* 1 day storage	*Maturity* matured stem *Storage* 4 days storage	[38]
-	Stem	Air dry; CO_2_-Soxhlet, maceration	Seremban Malaysia	CO_2_-Soxhlet: 43.81 mg RE/g dried material Maceration: 16.83 mg RE/g dried material	CO_2_-Soxhlet	Maceration	[28]

**Table 5:** Nutritional composition of *C. nutans.*

Sample details	Carbohydrate	Protein	Fat	Fiber	Ash	Moisture	Vitamin	Mineral	Reference
China- Leaf	-	5.73 ± 0.14%	0.50 ± 0.02%	2.71 ± 0.05%	-	78.30 ± 0.29%	C: 1.57 ± 0.07 mg/100 g B1: 0.27 ± 0.04 mg/100 g	-	[40]
Seremban Malaysia- Leaf	73.27 ± 3.14%	5.16 ± 0.08%	2.21 ± 0.66%	-	10.0 ± 0.20%	9.28 ± 0.40%		Potassium: 1097.90 ± 6.93 mg/100 g Calcium: 874.50 ± 31.25 mg/100 g Sodium: 6.78 ± 1.01 mg/100 g Copper: 0.26 ± 0.01 mg/100 g	[39]

**Table 6:** HPLC quantification of *C. nutans.*

Sample details	Stationary phase	Mobile phase	Detector; Wavelength	Flow rate (mL/min)	Elution method	Standard for Quantification	The corresponding concentration	Reference
Hot water; Oven dry	Thermo Scientific ODS Hypersil (5 *μ*m, 100 × 4.6 mm)	Water with acetic acid (pH 2.74), acetonitrile	UV; 272–370 nm	0.8	Gradient	Gallic acid, catechin, caffeic acid, **quercetin**	0.25 ± 0.01 *μ*g/mg of dried sample	[43]
Leaf;** Taiping (Perak)**, Kota Tinggi (Johor), Sendayan (Negeri Sembilan); Ethanolic; Oven dry, Sonication	Kinetex Pentafluoro-phenyl (PFP) (5 *μ*m, 250 × 4.6 mm)	Water with 0.8% (v/v) glacial acetic acid, acetonitrile	UV-Vis/DAD; 330 nm	0.7	Gradient	**Shaftoside**, orientin, isovitexin, vitexin	17.43 ± 0.01 mmol/g	[23]
**Buds**, Leaf; 1, **6** and 12 months old; Methanol; Freeze dry	C18 (5 *μ*m, 250 × 4.6 mm)	0.03 M *ortho*-phosphoric acid, methanol	UV-Vis; 260–360 nm	1.0	Gradient	Catechin, kaempferol, luteoli 7-*O*-*β*-D-glucoside, quercetin, **gallic acid **monohydrate, caffeic acid	5.963 ± 0.545 mg/g DW	[41]
Aerial; 86% ethanol; Oven dry; MAE, p-MAE, **SFE**	Symmetry C18 (5 *μ*m, 150 × 4.6 mm)	Methanol, 2-propanol	UV-ELSD; 210 nm	0.7	Isocratic	**β** **-sitosterol**	0.83 ± 0.10 mg/g DM	[30]
Leaf; **Hot water**, **water, 80**%** methanol**; Freeze dry; Sonication	LUNA C18 (5 mm, 250 × 4.6 mm)	Water with 6% acetic acid (pH 2.27), acetonitrile	DAD; 320 nm	0.5	Gradient	Cinnamic acid, **protocatechuic acid**, ferulic acid, gallic acid, *p*-coumaric, chlorogenic acid, vanillic acid, caffeic acid	33.28 mg/g extract	[42]
Leaf, Aqueous, **80**%** methanol**; Sonication	LUNA C18 (5 mm, 250 × 4.6 mm)	Water with 6% acetic acid (pH 2.27), acetonitrile	DAD; 320 nm	0.5	Gradient	Cinnamic acid, protocatechuic acid, ferulic acid, gallic acid, *p*-coumaric, **chlorogenic acid**, vanillic acid, caffeic acid	33.38 ± 0.31 mg/g extract	[39]

The text in **bold** word indicated that particular extract has the highest concentration of the particular standard.

**Table:** (a) Pharmacological activity: antivenom

Experiment design (Experiment model; Venom; Assay; Test subject)	Extract	Plant part	Plant Source	Extract dose; Route of administration	Result	Reference
*in vitro; Heterometrus laoticus *scorpion venom; Pre-incubated extract with 0.2 *μ*g/*μ*L venom-Cell lytic test incubated for 30 min; CEFs	Water	Leaf	Thailand	0.706, 0.406 mg/mL	Extract at 0.706 mg/mL give 46.51% of efficiency but the cytotoxic of extract is questionable	[65]
*in vitro; Apis mellifera *Linn. bee venom; Pre-incubated extract with 0.6 *μ*g/*μ*L venom-Cell lytic test incubated for 30 min; CEFs	Water, 90% and 50% ethanol	Leaf	Thailand	0.706, 0.406 mg/mL	Ineffective	[66]
*in vivo; Laticauda colubrine *snake venom; Mice and Mongrel dog	Water; Maceration	Leaf	Sarawak Malaysia	6 mg/kg per mouse (i.p), 20 mg/kg per dog (i.v)	Ineffective	[7]
*in vivo; Naja naja siamensis* snake venom; Isolated rat phrenic-nerve diaphragm preparations; Mice	Water	Leaf	Thailand	p.o, i.p	Ineffective	[5]
*in vivo; *Snake venom; Mice	Water	Leaf	Thailand	-	Reduce mortality rate from 100% to 63 ± 3.34%	[67]
*in vivo; *Snake venom; Mice	95% alcohol	Leaf	Thailand	2000 mg/kg; i.v, i.p, p.o	Ineffective	[67]

CEFs: Chick embryo fibroblasts.

**Table:** (b) Pharmacological activity: analgesic/antinociceptive

Experiment design (Experiment model; Assay; Test subject)	Extract	Plant part	Plant Source	Extract dose; Route of administration; positive control	Result	Reference
*in vivo*; Acetic acid-induced writhing test; Mice	Water, methanol, chloroform,* n*-butanol	Leaf	Thailand	Phenylbutazone (100 mg/kg)	Effective (*n*-butanol extract at 90 mg/kg)	[68, 69]
*in vivo*; Acetic acid-induced abdominal writhing test-Pre-treatment 1 h before test; ICR mice (adult male, 25–30 g)	100% Methanol; Oven dry; Soaking	Leaf	Kuala Lumpur Malaysia	100, 250, and 500 mg/kg; ASA (100 mg/kg)	ED_50_: 279.3 mg/kg	[19]
*in vivo*; Formalin-induced paw licking test-Pre-treatment 1 h before test; Sprague Dawley rat (adult male 150–180 g)	100% Methanol; Oven dry; Soaking	Leaf	Kuala Lumpur Malaysia	100, 250, and 500 mg/kg; ASA (positive, 100 mg/kg); morphine (5 mg/kg)	Early phase: ED_50_: >500 mg/kg Late phase: ED_50_: 227.7 mg/kg	[19]
*in vivo*; Hot plate test at 50°C- Pre-treatment 1 h before test; ICR mice (adult male; 25–30 g)	100% Methanol; Oven dry; Soaking	Leaf	Kuala Lumpur Malaysia	100, 250, and 500 mg/kg; morphine (5 mg/kg)	500 mg/kg significant delay response at the interval of 60 to 210 min	[19]
*in vivo*; Hot glass jar at 72°C water bath; Albino mice (either sex, 40–50 g)	95% Ethanol; Maceration	Leaf	Thailand	5 g/kg	Ineffective	[92]
*in vivo*; Hot water plate test; Mice	*n*-Butanol	Leaf	Thailand	p.o, i.p; morphine	Ineffective	[68, 69]

**Table:** (c) Pharmacological activity: anti-inflammatory

Experiment design (Experiment model; Assay; Test subject)	Extract	Plant part	Plant Source	Extract dose; Route of administration; Positive control	Result	Reference
*in vitro*; fMLP induced elastase release- Pre-treatment; Human neutrophils	Methanol; Air dry; Percolation	Whole plant	Thailand	0.01–100 *μ*g/mL; Indomethacin (1–100 *μ*g/mL)	IC_50_: 186.8 ± 20.5 *μ*g/mL elicited a weak, but significant inhibition of human neutrophil elastase release (100 nM)	[70]
*in vitro*; fMLP/CB induce elastase release; Human neutrophils	80% ethanol, ethyl acetate, *n*-hexane; Air dry; Soaking	Aerial	Taichung Taiwan	10 *μ*g/mL	80% ethanol showed highest inhibition: 68.33 ± 5.49%	[52]
*in vitro;* fMLP induced neutrophil superoxide anion generation (pre-incubation for 10 min)	Methanol; Air dry; Percolation	Whole	Thailand	0.01–100 *μ*g/mL; Indomethacin	IC_50_: 23.4 ± 3.1 *μ*g/mL	[70]
*in vitro*; Superoxide anion generation assay; Human neutrophils	80% ethanol, ethyl acetate, *n*-hexane; Air dry; Soaking	Aerial	Taichung Taiwan	10 *μ*g/mL	80% ethanol showed highest inhibition: 28.52 ± 2.55%	[52]
*in vitro*; fMLP induced neutrophil myeloperoxidase (MPO) production	Methanol; Air dry; Percolation	Whole	Thailand	0.01–100 *μ*g/mL; Indomethacin	IC_50_: 219.5 ± 25.7 *μ*g/mL Inhibition via reduced neutrophil migration	[70]
*in vitro*; Immunoblotting-LPS induced TLR-4 inflammatory proteins; Protein lysate from macrophage	Polar; Soaking	Leaf, stem	Pahang Malaysia	20 *μ*g/mL	Significantly reduced the LPS induced phosphorylation of p65, p38, ERK1/2, JNK1/2, IRF3	[62]
*in vitro;* LPS induced TLR-4 assay; HEK-Blue-hTLR4 cells	Polar, nonpolar; Soaking	Leaf, stem	Pahang Malaysia	100 *μ*g/mL	IC_50_: 21.3 ± 5.0 (leaf polar) to 29.4 ± 9.0 (leaf non-polar) *μ*g/mL	[62]
*in vitro*; LPS induced cytokine production assay-1 h pre-treatment; murine macrophages RAW 264.7 cell	Polar, nonpolar; Soaking	Leaf, stem	Pahang Malaysia	100 *μ*g/mL	Polar leaf (*p *< 0.05) inhibited TNF-*α*, IFN-*γ*, IL-1*β*, IL-6, IL12p40, IL-17 production	[62]
*in vitro*; LPS induced NO- 1 h pre-treatment- Griess assay; Murine macrophages RAW 264.7 cell	Polar, nonpolar; Soaking	Leaf, stem	Pahang Malaysia	100 *μ*g/mL	IC_50_: 18.9 ± 3.6 (leaf polar) to 43.1 ± 4.7 (leaf non-polar) *μ*g/mL	[62]
*in vivo*; Acetic acid induced vascular permeability model; Mice	Water, methanol, chloroform,* n*-butanol	Leaf	Thailand	Indomethacin (4 mg/kg)	Effective, *n*-butanol at 540 mg/kg as potent as indomethacin	[68, 69]
*in vivo*; EPP induced rat ear oedema model- pre-treatment; Sprague Dawley rats (male, 40–60 g)	Methanol; Air dry; Percolation	Whole plant	Thailand	3, 6, 9 mg/20 *μ*L acetone per ear; Apply topically; Indomethacin (2 mg/20 *μ*L)	At dose 9 mg EPP/ear: 79% oedema inhibition at 15 min, 44.4% MPO reduction after 120 min of induction	[70]
*in vivo*; Carrageenan induced paw oedema model-1 h pre-treatment; Sprague Dawley rats (male, 100–120 g)	Methanol; Air dry; Percolation	Whole plant	Thailand	50, 100, 200 mg/kg; p.o; Indomethacin (20 mg/kg)	200 mg/kg of extract inhibit 59% of oedema at 3 h	[70]
*in vivo*; Carrageenan induced paw oedema; Albino mice (40–50 g)	95% Ethanol; Maceration	Leaf	Thailand	5000 mg/kg; p.o	17.73% at 3 h and 36.47% at 6 h of oedema inhibition	[92]
*in vivo*; Carrageenan induced paw oedema model; Mice	*n*-Butanol	Leaf	Thailand	p.o; acetylsalicylic acid (100 mg/kg)	Effective*, n*-butanol at 270 mg/kg as potent as ASA	[68, 69]
*in vivo*; Granuloma pouch model; Wistar rats (male)	Aqueous ethanol cream	Leaf	Thailand	125 mg cream/rat; Apply topically; Prednisolone (0.25%)	Inhibit 48.3% granuloma formation	[71]
*in vivo*; Granuloma pouch model; Wistar rats (male)	95% ethanol cream	Leaf	Thailand	125 mg cream/rat; Apply topically; Prednisolone (0.25%)	Inhibit 50.1% granuloma formation	[71]
*in vivo*; Granuloma pouch model-1 h pre-treatment; Mice	*n*-Butanol cream	Leaf	Thailand	270 and 540 mg/kg; Apply topically	Ineffective	[68, 69]
*in vivo*; Granuloma pouch model; Wistar rats (male)	Cold cream	Leaf	Thailand	125 mg cream/rat; Apply topically; Prednisolone (0.25%)	Inhibit 50.98% granuloma formation	[71]

**Table:** (d) Pharmacological activity: immunomodulating effect

Experiment design (Experiment model; Assay; Incubation period; Test subject; Extract dose; Positive control)	Extract	Plant part	Plant Source	Result	Reference
*in vitro*; with and without fMLP induced Chemotaxis and Chemokinesis; 45 min; Human neutrophils; 0.1–100 *μ*g/mL; Indomethacin (0.01–100 *μ*g/mL, IC_50_: 56.3 ± 3.5 ng/ml)	Methanol; Air dry; Percolation	Whole plant	Thailand	Chemotaxis: With fMLP induction, IC_50_: 2.7 ± 0.6 *μ*g/mL, Without fMLP, IC_50_: >100 *μ*g/mL Chemokinesis: With fMLP induction, IC_50_: 5.5 ± 0.6 *μ*g/mL, Without fMLP, IC_50_: 5.0 ± 0.5 *μ*g/mL	[70]
*in vitro*; Apoptosis assessment via morphological, flow cytometry-Annexin V binding; 20 h; Human neutrophils; 10–500 *μ*g/mL; Dexamethasone (1 *μ*M, inhibited 54.5% neutrophil apoptosis)	Methanol; Air dry; Percolation	Whole plant	Thailand	No significant effect on neutrophil apoptosis	[70]
*in vitro*; ConA & LPS-induced IL-10 & TNF-*α* expression-Real time PCR analysis; 16 h; PBMC; 1.56 mg/mL	Methanol; Oven dry; Maceration	Leaf, Stem	Chiang Mai Thailand	Reduce IL-10 mRNA expression, no modulating effect on TNF-*α* mRNA expression	[72]
*in vitro*; IFN-*γ* expression Splenocyte from ovalbumin-primed BALB/c mice	80% Ethanol; Air dry; Soaking	Aerial	Taichung Taiwan	0.1 *μ*g/mL: upregulation of IFN-*γ*. 100 *μ*g/mL: down-regulation of IFN-*γ*	[52]
*in vitro*; HaCaT; IFN-*γ*/TNF-*α*-induced apoptosis- MTT assay; 1 and 100 *μ*g/mL	Ethanol; Maceration	Leaf	Thailand	Significantly (*p* < 0.05) suppressed keratinocytes apoptosis	[63]
*in vitro; *Lymphocyte proliferation assay-^3^H- thymidine-radioactivity; 72 h; HBMC; 0.5–5000 *μ*g/mL	Ethanol; Soxhlet	Leaf	Thailand	0.5–5 *μ*g/mL cause proliferation increase, 2.5, 5 mg/mL cause proliferation decrease	[73]
*in vitro; *NK activity-K562 as target cell-cytotoxicity assay; 72 h; HBMC; 0.5–5000 *μ*g/mL	Ethanol; Soxhlet	Leaf	Thailand	1 and 5 mg/mL-cause NK activity decrease	[73]
*in vitro; *IL-2 production-ELISA; 72 h; HBMC; 0.5–5000 *μ*g/mL	Ethanol; Soxhlet	Leaf	Thailand	undetectable IL-2	[73]
*in vitro; *IL-4 production-ELISA; 72 h; HBMC; 0.5–5000 *μ*g/mL	Ethanol; Soxhlet	Leaf	Thailand	2.5 and 5 mg/mL-increase IL4 production	[73]
*in vitro; *Lymphocyte subpopulation-flow cytometry assay; 72 h; HBMC; 0.5–5000 *μ*g/mL	Ethanol; Soxhlet	Leaf	Thailand	no change in the percentages of CD3^+^, CD4^+^, CD8^+^, CD16^+^/CD56^+^ and CD19^+^ cells	[73]

**Table:** (e) Pharmacological activity: neuroprotective and neuromodulating function

Experiment design (Experiment model; Assay; Incubation period; Test subject)	Extract	Plant part	Plant Source	Extract dose; Route of administration; Positive control	Result	Reference
*in vitro*; OGD–reoxygenation and hypoxic neuronal death, Cell viability assay*; *Mouse primary cortical neurons, cerebral astrocytes, cerebral endothelial cells	80% Ethanol; Soaking	Leaf	Singapore	5 *μ*g/mL	Extract suppressed post-hypoxic HDACs activation and reduce OGD-caused neuronal death	[75]
*in vitro*; OGD–reoxygenation; 24 h Human SH-SY5Y neuroblastoma cells	80% Ethanol; Soaking	Leaf	Singapore	100 *μ*g/mL	Extract modulated cPLA2 expression induction in SH-SY5Y cells by HDAC inhibitors, MS-275, MC-1568, TSA and inhibited HAT activity.	[74]
*in vitro; *OGD–reoxygenation; 12 h and 24 h; Mouse primary cortical neurons	80% Ethanol; Soaking	Leaf	Singapore	1.6, 6.25 *μ*g/mL	Extract inhibited levels of cPLA2 mRNA expression in primary cortical neurons subjected to 0.5 h OGD injury	[74]
*in vitro; *OGD; CCK-8; up to 12 h; neutron pre-treated with extract 1 h before OGD; Mouse primary cortical neurons from E15.5 Balb/c mouse embryos	80% Ethanol; Soaking	Leaf	Malaysia	6.25 *μ*g/mL	Extract treated neurons showed significant increment in cell viability	[76]
*in vitro; *OGD–reoxygenation; CCK-8, MMP, apoptosis analysis, transient transfection and chromatin reporter assay; 0.5 h OGD+ 4–24 h Reoxygenation; Neurons treated with extract (1 h before, on the onset or after OGD-reoxygenation); Mouse primary cortical neurons from E15.5 Balb/c mouse embryos	80% Ethanol; Soaking	Leaf	Malaysia	0.075–20 *μ*g/mL	Neurons treated with extract before, at the onset or after OGD showed increment in dose dependent manner but protective effect of extract was lesser when applied after OGD. Neurons treated with 6.25 *μ*g/mL extract at the onset of OGD has reduced MMP breakdown, apoptotic death, and pro-apoptotic (caspase-3, PARP-1) and has higher anti-apoptotic (14-3-3*ε*, p-Bad, Bcl-2) markers. Neurons treated with 2.5–10 *μ*g/mL extract at the onset of OGD suggested a dose dependent increase effect in PPAR-*γ* mRNA level.	[76]
*in vitro*; Anti-spasmotic effect on acetycholine induced rat's bladder tissue contraction; Isolated tissues of rat's bladder	Ethanol	Leaf, stem	Malaysia	Mebeverine hydrochloride and detrusitol	Stem at 120 mg/mL: 79.77% Leaf at 100 mg/mL: 26.62% Detrusitol at 200 *μ*g/mL: 89.86%, Mebeverine at 10 mg/mL: 80.74% of contraction inhibition	[93]
*in vivo*; Ellman assay-Acetylcholinesterase activity in brain, liver, kidney, heart; Pretreatment for 14 days (daily treatment (once)); Balb/C mice (male, 25 ± 5 g)	Methanol, Air dry, Maceration	Leaf	Seremban Malaysia	250, 500, 1000 mg/kg bw	All doses of extracts caused the higher acetylcholinesterase activity in liver, kidney, and heart compared to control group while they did not caused any difference in brain	[77]
*in vivo*; MCA occlusion stroke model; Reperfusion infarct volume, behaviour assessment, MRI; extract treated 1 h before, immediate or 3–24 h after 30 min MCA occlusion or 1 d after 15 min MCA acclusion; Long- Evans rats (male, 7-8 week-old)	80% Ethanol; Soaking	Leaf	Malaysia	10–60 pg (i.c.v., after 30 min MCA occlusion); 24 mg/kg body wt (i.p, 1 h before or 3–24 h after 30 min or 24 h after 15 min MCA occlusion)	I.C.V. treatment of 40 pg extract reduced apoptotic neuronal death, showed a maximal decrease of cerebral infarct volume at 1 day reperfusion. PPAR-*γ*, C/EBP*β* mRNA levels, anti- apoptotic 14-3-3*ε*, p-Bad, and Bcl-2 levels were upregulated. Pro-apoptotic cleaved PARP-1 and cleaved caspase 3 protein levels were downregulated. Extract improved the functional outcomes based on the behavior observation at day 7 after ischemia (elevated body swing test and Bederson's postural reflex task) and at day 14 after ischemia (better ladder rung walking test). Extract via i.p treatment also significantly reduced infarct volume except 24 h after 30 min MCA occlusion. Behavior test conducted also showed better functional outcomes and smaller infarct volume via MRI.	[76]

**Table:** (f) Pharmacological activity: antidiabetic and *α*-glucosidase inhibition activity

Experiment design (Experiment model; Assay; Test subject)	Test sample	Plant part	Plant Source	Extract dose; Route of administration; Positive control	Result	Reference
*in vitro; α*-glucosidase inhibition assay	Hot water; Oven dry	-	Malaysia	50 mg/mL	88.2% of inhibition	[37]
*in vitro; α*-glucosidase inhibition assay	Methanol; Oven dry; Sonication	Leaf, Stem	Seremban Malaysia	5000 *μ*g/mL (in stock)	13.57 (leaf), 17.67 (stem) % of inhibition	[61]
*in vitro; α*-glucosidase inhibition assay	70% Ethanol; Oven, air, freeze dry; Sonication, soaking	Leaf, stem	Seremban Malaysia	5000 *μ*g/mL (in stock); Quercetin	Lowest: 5.31% (Leaf-Freeze dry-Soaking), Highest: 41.70% (Leaf-Oven dry-sonication)	[4]
*in vitro; α*-glucosidase inhibition assay	CO_2_-Soxhlet, Maceration	Stem	Seremban Malaysia	5000 *μ*g/mL (in stock); Quercetin	CO_2_-Soxhlet: 95.79% of inhibition Maceration: 58.23% of inhibition	[28]
*in vivo*; Alloxan induced model- daily treatment for 9 days;* Swiss webster *mice (male)	Hot water; Sun dry	Leaf	Bandung Indonesia	50, 100, and 150 mg/kg BW; p.o; Oral glibenclamide	150 mg/kg significantly lower blood glucose serum level from 442 ± 149 mg/dl (day 0) to 195 ± 66 mg/dl (day 9)	[25]
*in vivo*; High fat and high cholesterol diet (HFHC) induced insulin resistance-7 weeks periods-daily treatment of extract; Sprague Dawleys rat (male, 200–250 g)	Water, 80% methanol; Sonication	Leaf	Seremban Malaysia	500, 250 or 125 mg/kg/day/rat; p.o.; Simvastatin	*C. nutans *attenuated the metabolic effects and transcriptional changes induced by the HFHC diet	[39]
*in vivo*; Glucose solution (p.o, 2 g/kg bw);* Swiss webster *mice (male)	Insoluble ethyl acetate fraction from ethanol	Leaf	Bandung Indonesia	100 mg/kg bw; p.o	Decreased 18.4% blood glucose serum level in 3 h	[25]

**Table:** (g) Pharmacological activity: antioxidant

Test sample	Plant part	Plant Source	Extract dose; Positive control	Result	Reference
(1) Type of assay: *in vitro*; DPPH scavenging assay					

Hot water; Oven dry	-	Malaysia	1 to 16 mg/mL	16 mg/mL: ~60% inhibition	[37]
Hot water; Unfermented and fermented; Microwave-oven dry, freeze dry; Infusion for 1, 2, 5, 10, 15, and 20 min	Leaf	Sabah Malaysia	0.1, 0.2, 0.4, 0.6, 0.8, 1.0 mg/mL	Unfermented possessed higher DPPH inhibition than fermented tea	[36]
Hot water, water, 80% methanol, methanol, ethyl acetate, hexane; Freeze dry; Sonication	Leaf, stem	Seremban Malaysia	Trolox	6.12% (hexane leaf) to 55.12% (80% methanol leaf) inhibition	[42]
Water, methanol, chloroform; Oven dry; Soaking	Leaf	Serdang Malaysia	12.5, 25, 50 and 100 *μ*g/mL; Trolox	Chloroform > methanol > water 864.11 ± 73.49 to 7852.63 ± 449.90 (chloroform) *μ*g Teq/g extract	[32]
Methanol; Sun dry	Aerial	Vietnam	0, 20, 40, 60, 80, 100 *μ*g/mL	IC_50_: 114.50 *μ*g/mL	[49]
Methanol; Air dry; Percolation	Whole	Thailand	1–400 *μ*g/mL; Trolox	IC_50_: 240.1 ± 15.3 mg/mL	[70]
Methanol; Air dry; Maceration	Leaf	Seremban Malaysia	0.25 to 10 mg/mL; Quercetin	IC_50_: 1.33 ± 0.0001 mg/mL	[24]
Methanol; Oven dry; Soaking	Leaf, stem	Seremban Malaysia	5000 *μ*g/mL (stock); Quercetin	Leaf: IC_50_: 1126.63 *μ*g/mL Stem: IC_50_: 1548.89 *μ*g/mL	[61]
Methanol; 1, 6 and 12 months old; Freeze dry	Bud, leaf	Serdang Malaysia	BHT, caffeic acid, *α*-tocopherol	IC_50_: 64.6 (1 year old buds) to 112.1 (1 year old leaf) *μ*g/mL	[41]
Methanol, Petroleum ether, Ethyl acetate; Oven dry; Soaking	Leaf, stem	Taiping Malaysia	0.2 to 10.0 mg/mL	4 mg/mL: leaf petroleum ether give 82% of DPPH inhibition	[88]
Methanol, ethyl acetate, chloroform, hexane	Whole	Jelebu Malaysia	1000 *μ*g/mL; BHT	50.50 ± 0.03% (Hexane) to 70.96 ± 0.03% (chloroform) inhibition	[53]
98% methanol, ethyl acetate, chloroform, hexane; Sun dry; Soaking	Stem	Vietnam	Vitamin C	Ineffective	[47]
50% ethanol; Soaking	Leaf	Thailand	1–300 *μ*g/mL; Ascorbic acid	IC_50_: 110.4 ± 6.59 *μ*g/mL	[94]
70% ethanol; Freeze dry; Sonication	Leaf	Perak Malaysia	200–1000 *μ*g/mL; Green tea	IC_50_ not determined	[64]
70% ethanol; Freeze dry, oven dry, air dry; Sonication, soaking	Leaf, stem	Seremban Malaysia	5000 *μ*g/mL (stock); Quercetin	15.44 ± 2.21% (Stem-freeze dry-soaking) to 44.31 ± 3.16% (Leaf-oven dry-sonication) inhibition	[4]
80% Ethanol; Young, old; Storage duration (1, 2, 3, and 4 days)	Leaf, stem	Malaysia	-	*Maturity*: 31.24% (matured stem) to 112.12% (young leaf) *Storage duration*: 31.88% (4 days) to 101.85% (1 day) inhibition	[38]
Ethanol; Maceration	Aerial	Bangkok Thailand	1 mg/mL	Ineffective	[78]
Ethyl acetate	Leaf	Indonesia	BHT	IC_50_: 178.40 mg/L	[95]
CO_2_-Soxhlet, Maceration	Stem	Seremban Malaysia	5000 *μ*g/mL (stock); Quercetin	CO_2_-Soxhlet: 98.92% inhibition Maceration: 63.00% inhibition	[28]

(2) Type of assay: *in vitro*; FRAP assay					

Hot water; Unfermented and fermented; Microwave-oven dry, freeze dry; Infusion for 1, 2, 5, 10, 15, and 20 min	Leaf	Sabah Malaysia	0.1, 0.2, 0.4, 0.6, 0.8, 1.0 mg/mL	250.70 ± 49 (fermented-microwave oven dry-20 min infusion) to 438.80 ± 94 (unfermented-freeze dry-10 min infusion) mg/L	[36]
Hot water, water, 80% methanol, methanol, ethyl acetate, Hexane; Freeze dry; Sonication	Leaf, stem	Seremban Malaysia.	Gallic acid	~45 (hexane stem) to ~148 mg (hot water leaf) GAE/g extract	[42]
Methanol, ethyl acetate, chloroform, hexane	Whole	Jelebu Malaysia	1000 *μ*g/mL; Ascorbic acid	hexane and ethyl acetate (not determined) to 56.49 ± 0.05% (methanol)	[53]
Methanol; 1, 6 and 12 months old; Freeze dry	Bud, leaf	Serdang Malaysia	BHT, caffeic acid, Vitamin C	209 *μ*M of Fe (II)/g (1-month-old leaves) to 488 *μ*M of Fe (II)/g (6-month-old buds).	[41]
70% Ethanol; Freeze dry; Sonication	Leaf	Perak Malaysia	200–1000 *μ*g/mL; Green tea	very low absorbance (ineffective)	[64]
50% Ethanol; Soaking	Leaf	Thailand	1–100 *μ*g/mL; Ascorbic acid	17 mg ascorbate/g extract	[94]

(3) Type of assay: *in vitro*; Hydrogen peroxide scavenging activity					

Water, methanol, chloroform; Oven dry; Soaking	Leaf	Serdang Malaysia	12.5, 25, 50 and 100 *μ*g/mL; Quercetin	Methanol: highest radical scavenging: ~34% at 100 *μ*g/mL	[32]

(4) Type of assay: *in vitro*; Metal chelating activity					

Hot water; Oven dry	-	Malaysia	1, 2, 5, 10 mg/mL	10 mg/mL: ~90% inhibition	[37]

(5) Type of assay: *in vitro*; Nitric oxide scavenging assay					

Hot water; Oven dry	-	Malaysia	1, 5, 10 mg/mL	10 mg/mL: ~90% inhibition	[37]
Water; methanol, chloroform; Oven dry; Soaking	Leaf	Serdang Malaysia	12.5, 25, 50 and 100 *μ*g/mL	Water: 32.33% at 100 *μ*g/mL	[32]

(6) Type of assay: *in vitro*; ABTS cation radical scavenging assay					

Hot water; unfermented and fermented; microwave-oven dry, freeze dry; hot boiling water (100°C); infusion for 1, 2, 5, 10, 15, and 20 min	Leaf	Sabah Malaysia	0.1, 0.2, 0.4, 0.6, 0.8, 1.0 mg/mL	50.36 ± 4.07 (fermented-microwave oven dry-10 min infusion) to 74.03 ± 2.26 (unfermented-freeze dry-5 min infusion) mg AEAC/L	[36]
Hot water, water, 80% methanol, methanol, ethyl acetate, hexane; Freeze dry; Sonication	Leaf, stem	Seremban Malaysia	Trolox	18 (hexane leaf) to 65 (80% methanol-leaf) mg TE/g extract	[39]

(7) Type of assay: *in vitro*; Galvinoxyl radical scavenging activity					

Water; methanol, chloroform; Oven dry; Soaking	Leaf	Serdang Malaysia	12.5, 25, 50 and 100 *μ*g/mL; Trolox	Chloroform > methanol > aqueous Chloroform: 12248.82 *μ*g Teq/g extract	[32]

(8) Type of assay: *in vitro*; Superoxide radical scavenging activity					

Methanol; Air dry; Percolation	Whole	Thailand	Direct EPR scavenging effect	showed significant direct scavenging activity when the incubation time extended to 60 min	[70]
70% ethanol; Freeze dry; Sonication	Leaf	Perak Malaysia	Green tea; by NBT method	3651.5 U/g	[64]

(9) Type of assay: *in vitro*; Phorbol 12-myristate 13-acetate (PMA) induced peroxide production in rat macrophages					

50% Ethanol; Soaking	Leaf	Thailand	10, 30, 100 and 300 *μ*g/mL; Tiron	Fluorescent intensity: 58.72 ± 5.52 (30 *μ*g/mL), 51.92 ± 8.49 (100 *μ*g/mL), 53.50 ± 6.17 (300 *μ*g/mL)	[94]

(10) Type of assay: *in vitro*; Protective effect against peroxyl radicals initiator (AAPH)-induced oxidative hemolysis					

50% Ethanol; Soaking	Leaf	Thailand	200–1000 *μ*g/mL; Ascorbic acid	IC_50_: 359.38 ± 14.02 *μ*g/mL	[94]

(11) Type of assay: *in vivo*; Hyperlipidemia-associated oxidative stress model					

Water, 80% methanol; Freeze dry; Sonication	Leaf, stem	Seremban Malaysia	125, 250, 500 mg/kg; Sprague Dawley rats (male, 200–250 g); daily treatment for 49 days; p.o; Simvastatin	Both leaf extracts reduce oxidative stress through increasing serum antioxidant enzymes activity and upregulating the expression of hepatic antioxidant genes	[42]

**Table:** (h) Pharmacological activity: antimicrobial-antiviral

Experiment design (Experiment model; Virus strain; Assay; Test subject; Route of administration/Prescription)	Extract	Plant part	Plant Source	Extract dose; Positive control	Result	Reference
Type of virus: Varicella zoster virus (VZV)						

*in vitro*; VZV Kawaguchi strain; (1) DNA hybridization technique, (2) plaque reduction assay-pre, post, direct; WI-38 HEL cells	Organic; Soxhlet	Leaf	Thailand	Acyclovir IC_50_: 107 *μ*M (pre), 5 *μ*M (post), 30 *μ*M (direct)	Most effective: Direct inactivation (1) IC_50_: 1 : 2000 (pre), 1 : 6000 (post), >1 : 18000 (direct) (2) IC_50_: 1 : 2000 (pre), 1 : 4800 (post), 1 : 9600 (direct)	[80]
Clinical; Herpes zoster (shingles); Double-blinded, randomized trials; 60 patients; Apply topically 5 times daily for 7 to 14 days	5% extract cream	-	Thailand	Placebo	Lesion crusting within 3 days: *C. nutans *(89.3%), Placebo (0%) Lesion healing within 7 days:* C. nutans *(100%), Placebo (100%) Pain scores: *C. nutans *better than placebo group	[8]
Clinical; Herpes zoster (shingles); Randomized trials; 48 patients; Apply topically 5 times daily for 5 days	5% extract cream	-	Thailand	Placebo, Acyclovir	Lesion crusting within 3 days: *C. nutans *(81.3%), Acyclovir (27.2%), Placebo (0%) Lesion healing within 7 days:* C. nutans *(81.30), Acyclovir (18.20%), Placebo (0%)	[82]
Clinical; Herpes zoster (shingles); Double-blinded, block randomization; 120 patients; Apply topically 3 times daily from 1 to 26 days	120 patients	-	Thailand	Placebo	Lesion crusting within 3 days: *C. nutans *(27.5%), Placebo (8.6%) Lesion healing:* C. nutans *(14 days) Placebo (18 days) Symptom reduction: *C. nutans* better thanplacebo groups	[81]

Type of virus: Herpes simplex virus (HSV)						

*in vitro*; HSV-1-KOS strain; Plaque reduction assay-post; Vero cell	Methanol, dichloromethane,* n*-hexane; Soxhlet	Leaf	Thailand	12.5–100 *μ*g/mL; Acyclovir (IC_50_: 0.09 *μ*g/mL)	IC_50_: 32.05 ± 3.63 (*n*-hexane) to 64.93 ± 7.00 (methanol) *μ*g/mL	[83]
*in vitro*; HSV-1F strain; Plaque reduction assay-pre, post; Vero cell	Ethyl acetate; Oven dry; Soxhlet	Leaf	Thailand	19 *μ*g/mL; Acyclovir (5 *μ*g/mL, post), Dextran sulfate (2 *μ*g/mL, pre)	Pre: IC_50_: 7.6 *μ*g/mL significantly reduce plaques Post: ineffective	[79]
*in vitro*; HSV-1 strain; Plaque reduction assay-pre, post-incubated for 48 h; Vero cell	Chloroform; Soxhlet	Leaf	Thailand	Acyclovir (IC_50_: 0.64 ± 0.07 *μ*g/mL)	Pre: less than 50% inhibition of plaque formation Post: IC_50_: 115.00 *μ*g/mL	[56]
*in vitro*; HSV-2-strain G, 5 clinical HSV-2 isolates; Plaque reduction assay (post), Yield reduction assay (post), Inactivation kinetics (direct, 4 h); Vero cell	Methanol; Air dry; Percolation	Whole	Thailand	2500 *μ*g/mL	Ineffective	[96]
*in vitro*; HSV-2-Baylor 186; Plaque reduction assay-post; Vero cell	Methanol, dichloromethane,* n*-hexane; Soxhlet	Leaf	Thailand	12.5-100 *μ*g/mL; Acyclovir (IC_50_: 0.43 *μ*g/mL)	IC_50_: 65.13 ± 2.22 (methanol) to 72.62 ± 12.60 (n-hexane) *μ*g/mL	[83]
*in vitro*; HSV-2 standard strain; Plaque reduction assay-pre, post, direct; BHK cell	Ethanol (A2, C1, C2, C3, C4, C5 fraction)	Leaf	Thailand	-	C2, C3, C4: 1 : 2400 dilution cause 100% plaque inhibition, through extracellularly pathway	[97]
*in vitro*; HSV-2 strain; Plaque reduction assay-pre, post-incubated for 96 h; Vero cell	Chloroform; Soxhlet	Leaf	Thailand	Acyclovir (IC_50_: 0.80 *μ*g/mL)	Post: IC_50_: 140.00 ± 3.00 *μ*g/mL	[56]
Clinical; HSV-2-Herpes genitalis; Sequential randomisation; 77 patients; Apply topically 4 times daily for 6 days	5% extract cream	-	Thailand	Placebo, Acyclovir (Zovirax)	Lesion crusting within 3 days: *C. nutans *(98.6%), Acyclovir (80.8%), Placebo (12.5%) Lesion healing within 7 days:* C. nutans *(88.9%), Acyclovir (84.6%), Placebo (20.8%) *C*.* nutans *has no sticky, burning, stinging pain and side effects.	[82, 84]
Clinical; HSV-2-Herpes genitalis; Randomized trials; 163 patients; Apply topically 4 times daily for 6 days	5% extract cream	-	Thailand	Placebo, Acyclovir	The lesion crusting and healing speed were significantly better in the *C. nutans *and acyclovir treated groups compared to the placebo groups. No side effect observed *C. nutans *treated group.	[85]

Type of virus: Fish pathogenic virus						

*in vitro*; IHNV, OMV, IPNV strain; Plaque reduction assay-pre, post, direct; CHSE-214 cells	Ethanol; Soxhlet	-	Thailand	100 *μ*g/mL (pre, post), 500 *μ*g/mL (direct)	Direct: 100% (IHNV and OMV), 0% (IPNV) of plaque reduction Pre: 31% (IHNV), 54% (OMV), 74% (IPNV) of plaque reduction Post: 25% (IHNV), 48% (OMV), 3% (IPNV) of plaque reduction	[98]

Type of virus: Crustaceans (shrimp and prawn) infectious virus						

*in vivo; *YRV-RNA virus; Anti-viral test-direct; cultured black tiger shrimp	Ethanol; Soxhlet	Leaf	Thailand	0.1 to 10 mg/mL	Minimum inhibition: 1 *μ*g/mL	[87]
*in vivo;* YRV-RNA virus; Protective efficacy assay-14 days observation; daily twice treatment for 7 days-pre; cultured black tiger shrimp; p.o	Ethanol; Soxhlet	Leaf	Thailand	0, 0.1, 1 and 10 g/kg of pellet	1 g/kg of feed exhibited best protective efficacy with 57.6%	[87]

Type of virus: Mosquito-borne virus						

*in vitro*; DENV-2 strain 16681; Western blot assay, ECL detection kit-post; Naïve Huh-7 cells	80% Ethanol; Air dry; Soaking	Aerial	Taichung Taiwan	Ribavirin	IC_50_: 31.04 *μ*g/mL	[52]

Type of virus: Poultry and bird contagious virus						

*in vitro; *NDV- La Sota strain; Hemagglutination test-pre, post; CEFs	Water, Ethanol; Soxhlet	-	Thailand	31.25 g/mL (final)	Ineffective	[86]

**Table:** (i) Pharmacological activity: Anti-microbial-anti-bacterial

Experiment design (Experiment model; Bacteria strain; Assay)	Extract	Plant part	Plant Source	Extract dose; Positive control	Result	Reference
*in vitro*;* A. hydrophila;* Agar dilution assay	Ethanol; Soxhlet	-	Thailand	0 to 10.0 mg/mL	MIC: >10 mg/mL	[99]
*in vitro; B. cereus;* Disc diffusion assay	70% Methanol; Air dry; Soxhlet	Leaf	Perak Malaysia	25, 50 and 100 mg/mL; Ciprofloxacin	At 100 mg/mL: 15.00 ± 1.00 mm	[22]
*in vitro*;* B. cereus*; Microdilution assay	Methanol; Maceration	Leaf	Seremban Malaysia	0.1 to 12.5 mg/mL; Erythromycin, chloramphenicol	MIC: >12.5 mg/mL	[6]
*in vitro*; *B. cereus; *Microdilution assay	Ethyl acetate and its fraction; Oven dry; Soaking	Leaf, stem	Taiping Malaysia	0.08 to 5 mg/mL (final); Ampicillin	MIC: Ethyl acetate: 6.31 mg/mL, F7: 1.39 mg/mL	[88]
*in vitro*; *B. subtilis*; Light mediated disk diffusion assay	95% Ethanol; Soaking	Leaf	Thailand	5 mg/mL; Gentamycin	Ineffective	[89]
*in vitro*; *E. coli;* Microdilution assay	Hot water; Oven dry	-	Malaysia	0.4 to 50.0 mg/mL (final); Ampicillin	MIC: >50 mg/mL	[43]
*in vitro; E. coli;* Disc diffusion assay	70% Methanol; Air dry; Soxhlet	Leaf	Perak	25, 50 and 100 mg/ml; Ciprofloxacin	At 100 mg/ml: 17.00 ± 2.00 mm	[22]
*in vitro*;* E. coli*; Microdilution assay	Methanol; Maceration	Leaf	Seremban Malaysia	0.1 to 12.5 mg/mL; Erythromycin, chloramphenicol	MIC: 12.5 mg/mL	[6]
*in vitro*; *E. coli; *Microdilution assay	Ethyl acetate and its fraction; Oven dry; Soaking	Leaf, stem	Taiping Malaysia	0.08 to 5 mg/mL (final); Ampicillin	MIC: Ethyl acetate: >100 mg/mL, F7: 1.39 mg/mL	[88]
*in vitro*; *E. coli *DC10; Light mediated disk diffusion assay	95% Ethanol; Soaking	Leaf	Thailand	5 mg/mL; Gentamycin	Ineffective	[89]
*in vitro*; *E. coli *(wild); Light mediated disk diffusion assay	95% Ethanol; Soaking	Leaf	Thailand	5 mg/mL; Gentamycin	Ineffective	[89]
*in vitro*; *M. luteus;* Microdilution assay	Hot water; Oven dry	-	Malaysia	0.4 to 50.0 mg/mL (final); Ampicillin	MIC: >50 mg/mL	[43]
*in vitro*; MRSA*; *Disc diffusion assay, microdilution assay	Ethanol; Oven dry; Maceration	-	Thailand	5 mg/mL	No detected inhibition zone, MIC and MBC: >5 mg/mL	[100]
*in vitro*; MSSA K147; Light mediated disk diffusion assay	95% Ethanol; Soaking	Leaf	Thailand	5 mg/mL; Gentamycin	Ineffective	[89]
*in vitro*; *N. gonorrhoeae* & 11 clinical isolates; Disc diffusion	Methanol; Maceration	Leaf	Thailand	44 mg/mL (final); Norfloxacin	Ineffective	[101]
*in vitro*; *P. acnes*; Microdilution assay	Methanol; Maceration	Leaf	Seremban Malaysia	0.1 to 12.5 mg/mL; Erythromycin, chloramphenicol	MIC: >12.5 mg/mL	[6]
*in vitro*;* P. acnes;* Disc diffusion, microdilution assay	Ethanol; Maceration	Aerial	Thailand	Mangostin	MIC and MBC: >5 mg/mL	[102]
*in vitro*; *P. aeruginosa;* Microdilution assay	Hot water; Oven dry	-	Malaysia	0.4 to 50.0 mg/mL (final); Ampicillin	MIC: >50 mg/mL	[43]
*in vitro; P. aeruginosa;* Disc diffusion assay	70% Methanol; Air dry; Soxhlet	Leaf	Perak	25, 50 and 100 mg/ml; Ciprofloxacin	At 100 mg/ml: 13.00 ± 1.00 mm	[22]
*in vitro*; *P. aeruginosa *187 (wild); Light mediated disk diffusion assay	95% Ethanol; Soaking	Leaf	Thailand	5 mg/mL; Gentamycin	Ineffective	[89]
*in vitro*; *S. aureus;* Microdilution assay	Hot water; Oven dry	-	Malaysia	0.4 to 50.0 mg/mL (final); Ampicillin	MIC: >50 mg/mL	[43]
*in vitro; S. aureus;* Disc diffusion assay	70% Methanol; Air dry; Soxhlet	Leaf	Perak	25, 50 and 100 mg/ml; Ciprofloxacin	At 100 mg/mL: 26.67 ± 3.51 mm	[22]
*in vitro*; *S. aureus*; Microdilution assay	Methanol; Maceration	Leaf	Seremban Malaysia	0.1 to 12.5 mg/mL; Erythromycin, chloramphenicol	MIC: 12.5 mg/mL	[6]
*in vitro*; *S. aureus; *Disc diffusion assay, microdilution assay	Methanol; Sun dry; Maceration	Aerial	Vietnam	125–1000 mg/mL; Erythromycin (10–50 *μ*g/*μ*L)	16.67 mm inhibition zone, MIC: 62.5 mg/mL	[49]
*in vitro*; *S. aureus; *Disc diffusion assay, microdilution assay	Ethanol; Oven dry; Maceration	-	Thailand	5 mg/mL	No detected inhibition zone, MIC: 5 mg/mL, MBC: >5 mg/mL	[100]
*in vitro*; *S. enterica *serovar Paratyphi C; Disc diffusion assay	Distilled water, 70% ethanol, absolute ethanol, chloroform	Leaf	Malaysia	-	Showed inhibition zone	[26]
*in vitro*; *S. enterica *serovar Paratyphi B; Disc diffusion assay	Distilled water, 70% ethanol, absolute ethanol, chloroform	Leaf	Malaysia	-	Chloroform: a larger inhibition zone compare to other solvents.	[26]
*in vitro*; *S. enterica *serovar Typhi; Disc diffusion assay	Distilled water, 70% ethanol, absolute ethanol, chloroform	Leaf	Malaysia	-	Least inhibition zone	[26]
*in vitro*; *S. enterica *serovar Typhimurium; Disc diffusion assay	Distilled water, 70% ethanol, absolute ethanol, chloroform	Leaf	Malaysia	-	Showed inhibition zone	[26]
*in vitro*; *S. enterica Typhimurium; *Disc diffusion, microdilution assay	Methanol; Sun dry; Maceration	Aerial	Vietnam	125–1000 mg/mL; Erythromycin (10–50 *μ*g/*μ*L)	15.67 mm inhibition zone, MIC: 125 mg/mL	[49]
*in vitro*; *S. enterica Typhimurium; *Microdilution assay	Ethyl acetate and its fraction; Oven dry; Soaking	Leaf, stem	Taiping Malaysia	0.08 to 5 mg/mL (final); Ampicillin	MIC: Ethyl acetate: >100 mg/mL, F7: 1.39 mg/mL	[88]
*in vitro*; *S. enterica *serovar Weltevreden; Disc diffusion assay	Distilled water, 70% ethanol, absolute ethanol, chloroform	Leaf	Malaysia	-	Showed inhibition zone	[26]
*in vitro*;* S. epidermidis*; Microdilution assay	Methanol; Maceration	Leaf	Seremban Malaysia	0.1 to 12.5 mg/mL; Erythromycin, chloramphenicol	MIC: >12.5 mg/mL	[6]
*in vitro*;* S. epidermidis;* Disc diffusion, microdilution assays	Ethanol; Maceration	Aerial	Thailand	Mangostin	MIC and MBC: >5 mg/mL	[102]
*in vitro*; *S. epidermidis; *Disc diffusion, microdilution assays	Ethanol; Oven dry; Maceration	-	Thailand	5 mg/mL	No detected inhibition zone, MIC and MBC: 5 mg/mL	[100]
*in vitro*;* Streptococcus *sp.*;* Agar dilution assay	Ethanol; Soxhlet	-	Thailand	0 to 10.0 mg/mL	MIC: >10 mg/mL	[99]
*in vitro*; *V. harveyi;* Agar dilution assay	Ethanol; Soxhlet	-	Thailand	0 to 10.0 mg/mL	MIC: >10 mg/mL	[99]
*in vitro*; *V. paraliaemolyticlls;* Agar dilution assay	Ethanol; Soxhlet	-	Thailand	0 to 10.0 mg/mL	MIC: >10 mg/mL	[99]

**Table:** (j) Pharmacological activity: antimicrobial-antifungal

Experiment design (Experiment model; Fungal strain; Assay)	Extract	Plant part	Plant Source	Extract dose; Positive control	Result	Reference
*in vitro*; *A. fumigatus;* Light mediated disk diffusion assay	95% Ethanol; Soaking	Leaf	Thailand	5 mg/mL; Nystatin	Ineffective	[89]
*in vitro*; *C. albicans;* Light mediated disk diffusion assay	95% Ethanol; Soaking	Leaf	Thailand	5 mg/mL; Nystatin	Ineffective	[89]
*in vitro*; *C. albicans;* Microdilution assay	Ethyl acetate & its fraction; Oven dry; Soaking	Leaf, stem	Taiping Malaysia	0.08 to 5 mg/mL (final); Amphotericin B	Ethyl acetate: 6.31 mg/mL, F7: MIC: 1.39 mg/mL	[88]

**Table:** (k) Pharmacological activity: anticancer

Experiment design (Experiment model; Cancer cell(s); Incubation period; Assay; Test subject; Route of administration)	Extract	Plant part	Plant Source	Extract dose; Positive control	Result	Reference
*in vitro;* Mutated Salmonella typhimurium (T98 and T100) without metabolic activation; 6 days; Mutagenicity assay-Ames test	Water; Air dry; Maceration	Leaf	Penang Malaysia	500 *μ*g/well	Non-mutagenic activity in *S. typhimurium *histidine auxotrophs	[103]
*in vitro*; D24, MM418C1, MCF7, BT474 cancer cells; 24 h and 72 h; CCK-8 assay	Hot water, cold water, methanol, ethanol, dichloromethane	Leaf	Seremban Malaysia	2 mg/mL	D24 cell: cold water at 72 h caused 42.9% cell death (EC_50_: 1.63 mg/mL) MM418C1 and MCF7 cells: hot and cold water showed cytotoxicity BT474 cells: extract ineffective	[18]
*in vitro*; K562, HCT 116 cancer cells; MTT cytotoxicity assay	Water, 50% methanol, 100% methanol, 50% ethanol, 100% ethanol	Leaf	Malaysia	100 and 200 *μ*g/mL	Ineffective	[104]
*in vitro*; HepG2, IMR-32, NCI-H23, SNU-1, LS-174T, K-562, HeLa, Raji cancer cells; 72 h; MTT cytotoxicity assay	Water, ethanol, chloroform; Oven dry; Soaking	Leaf	Serdang Malaysia	3.125 to 100 *μ*g/mL	Most effective: chloroform with IC_50_: 47.70 *μ*g/mL (K562), 47.31 *μ*g/mL (Raji)	[32]
*in vitro*; Cultured Saos-2 human steosarcoma cells; HIF activity, MTT cytotoxicity assay	Methanol	Leaf	Malaysia	125–2000 *μ*g/mL	Ineffective	[105]
*in vitro*; HeLa cancer cells; 72 h; MTT cytotoxicity assay	Methanol; Freeze dry	Leaf, buds	Serdang Malaysia	10, 20, 40, 80, 160, 320 *μ*g/mL; Tamoxifen	Most effective: 6-month bud with IC_50_: 56.8 *μ*g/mL	[41]
*in vitro*; D24 melanoma cells; 24 h and 72 h, CCK-8 assay	Methanol; Air dry; Soaking with shaker	Leaf	Malaysia, Vietnam, Thailand	0–2 mg/mL (stock)	Chiang Dao Thailand extract has highest cytotoxicity: EC_50_: 0.95 mg/mL (24 h), 0.77 mg/mL (72 h)	[106]
*in vitro*; HepG2, MCF-7, NCI-H460, HeLa cancer cells; 48 h; Sulforhodamine B colorimetric assay	Sub-fraction F-III from Methanol extract	Stem	Vietnam	Camptothecin	Effective, IC_50_: 36.80 (HepG2), 57.36 (NCI-H460), 66.57 (MCF-7), 91.08 (Hela) *μ*g/mL	[47]
*in vitro; *HepG2 cell; 24 h; MTT cytotoxicity assay	Methanol, ethyl acetate, hexane, chloroform fraction	-	Malaysia	0, 6.25, 12.5, 25, 50, 100 *μ*g/mL (stock)	IC_50_: 43.93 (methanol), 55.61 (chloroform), 62.06 (ethyl acetate), 68.38 (hexane) *μ*g/mL	[35]
*in vitro; *MCF-7, HeLa cells, 72 h; MTT cytotoxicity assay	Methanol, ethyl acetate; Freeze dry; Soaking	Root	Sabah Malaysia	10–50 *μ*g/mL; Camptothecin (0.35 *μ*g/mL)	MCF-7 cell: IC_50_: 35.0 *μ*g/mL (methanol), 30.0 (ethyl acetate) *μ*g/mL HeLa cell: ~25% of inhibition for both extracts	[31]
*in vitro*; HeLa, K-562 cells; 24 h and 72 h; MTT cytotoxicity assay	Methanol, ethyl acetate, petroleum ether; Oven dry; Soaking	Leaf, stem	Taiping Malaysia	0.2–10.0 mg/mL	Petroleum ether leaf extract showed the strongest cytotoxic activity after 72 h, CC_50_: 18.0 *μ*g/mL (HeLa), 20.0 *μ*g/mL (K-562)	[88]
*in vivo*; MMS induced *Allium cepa* chromosome assay-post and suppressive treatment;* Allium cepa*	Water, methanol; oven dry	Leaf	Penang Malaysia	100, 200, 400, 800 mg/kg	Extract has repairing and anti-mutagenic effect	[90]
*in vivo*; Hepatocarcinoma tumor-bearing mice-daily treatment for 10 days; ICR mice (with normal T/B cells, 6–8 weeks, 18–22 g); Gastric probe	30% Ethanol fraction	Aerial	Seremban Malaysia	3 and 10 mg/kg; Fluorouracil (20 mg/kg)	8.2% (3 mg/kg) and 58.6% (10 mg/kg) of tumor size and weight reduction, higher reduction rate than positive control (37.1%)	[33]

**Table:** (l) Pharmacological activity: wound healing ability

Experiment design (Experiment model; Assay; Test subject)	Extract	Plant part	Plant Source	Extract dose	Result	Reference
*in vitro*; Migration rate of HGF at 0 h, 2 h, 4 h, and 6 h after scratching; Human gingival fibroblast (HGF)	Chloroform, Hexane; Sun dry	Leaf	Indonesia	10, 25, 50 and 100 *μ*g/mL	Culture supplemented with 10 *μ*g/mL of chloroform extract give the fastest HGF migration rate and wound recovery	[11]

**Table:** (m) Pharmacological activity: protective effect on plasmid DNA

Experiment design (Experiment model; Assay; Test Subject)	Extract	Plant part	Plant Source	Extract dose	Result	Reference
*in vitro*; DNA integrity assay; Supercoiled plasmid DNA react with O_2_^•^-generated via a riboflavin photoreaction treatment	70% Ethanol; Freeze dry; Sonication	Leaf	Perak Malaysia	10000, 1000, 100, 10 and 0 *μ*g/mL; Green tea	Extract reduced DNA cleavages, retained high levels of super-coiled plasmid DNA integrity and exhibited protection up to 50 min	[64]

**Table:** (n) Pharmacological activity: lipid elevated inhibition activity

Experiment design (Experiment model; Assay)	Extract	Plant part	Plant Source	Extract dose; Positive control	Result	Reference
*in vitro*; Porcine pancreatic lipase inhibition assay	Methanol; Freeze dry; Sonication	Leaf	Malaysia	100 *μ*g/mL; Orlistat	Ineffective, −22.56% indicates a promotion of pancreatic lipase activity	[21]

**Table:** (o) Pharmacological activity: oral mucositis and stomatitis treatment

Experiment design (Experiment model; Infection; Assay; Test subject; Route of administration/Prescription)	Extract	Plant Source	Positive control	Result	Reference
Clinical; Radiation induced oral mucositis; Single-blinded-Randomized trial; 60 patients; Apply 2 drops drip into mouth or on the lesion 3 to 5 times daily from first to last day of radiation	Glycerin payayor	Thailand	Benzydamine hydrochloride (Difflam, 3M, Australia)	The time onset of oral mucositis in the payayor group was significantly later, and its severity and pain score were less than those of the benzydamine group throughout the study period.	[10]
Clinical; Reccurent aphthous stomatitis; Double-blinded-controlled trial; 43 patients; apply to the ulcer 4 times daily	*C. nutans* in orabase	Thailand	Triamcinolone acetonide, placebo	*C. nutans* in orabase provide better healing of the ulcer as compared to placebo but efficacy was lesser compared to triamcinolone acetonide in orabase	[91]

**Table 8:** Pharmacological activity of isolated *C. nutans *compounds.

Experiment design (Experiment model; Assay; Incubation period; Test subject; Extract dose; Positive control)	Isolated compound	Result	Reference
(a) Anti-inflammatory activity			

*in vitro*; Prostaglandin E2 determination assay by the radioimmunoassay Immortalized COX-1 and COX-2 mouse lung fibroblast cell; Aspirin (IC_50_: 2.06 *μ*g/mL (COX-1), 3.57 *μ*g/mL (COX-2))	Cerebrosides	Ineffective	[55]

(b) Immunomodulating activity			

*in vitro*; ConA-induced T cell, LPS-induced B cell, evaluation of Th1 cytokines (IL-2 and IFN-*γ*), Th2 cytokines (IL-4 and IL-10); Splenocytes; Control on Th2 cytokines IL-4 (138.3 pg/mL) and IL-10 (1234.9 pg/mL)	(1) CN1 (shaftoside) (2) CN2 (stigmasterol) (3) CN3 (*β*-sitosterol) (4) CN4 (lupeol)	CN3 inhibit T lymphocyte proliferation the most (RP: 0.16) followed by CN2 (RP: 0.47), only CN1 inhibit B cell proliferation (RP: 0.77), all ineffective on Th1 cytokines, CN3 inhibit secretion of IL-4 (22.6 pg/mL) and IL-10 (63.9 pg/mL), CN3 significantly reduce activated helper T cells (54.3%) and activated cytotoxic T cells (62.2%)	[45]

(c) Anti-oxidant activity			

*in vitro;* DPPH scavenging assay; Vitamin C (IC_50_: 22.589 *μ*g/mL)	(1) Stigmasterol-*β*-*D*-glucoside (2) 3-amino-4,5-dihydroxyfuran-2(3H)-one	Compound 1: Ineffective Compound 2: IC_50_: 102.949 *μ*g/mL	[49]
*in vitro;* DPPH scavenging assay, FRAP assay; 1000 *μ*g/mL (stock); BHT (DPPH), Ascorbic acid (FRAP)	(1) Clinamide D (2) Clinamide E	(1) 76.05 ± 0.02%, (2) 72.84 ± 0.01% of DPPH inhibition, (1) 15.47 ± 0.03%, (2) 38.56 ± 0.02% of FRAP inhibition	[53]

(d) Anti-viral activity			

*Anti HSV-1 assay* *in vitro*; HSV-1 virus strain; Vero cell; Acyclovir (IC_50_: 2–5 *μ*g/mL)	Cerebrosides	Ineffective	[55]
*Anti HSV-1 assay* *in vitro*; HSV-1F strain; Plaque reduction assay-direct, pre, post; 72 h; Vero cell; Acyclovir, Dextran sulfate (1 mg/mL)	(1) 13^2^-hydroxy-(13^2^-R)-phaeophytin b (2) 13^2^-hydroxy-(13^2^-S)-phaeophytin a (3) 13^2^-hydroxy-(13^2^-R)-phaeophytin a	Direct: All exhibited 100% inhibition IC_50_: 1.96 nM, 3.11 nM, and 3.11 nM, respectively, Post: 30% of inhibition	[107]
*Anti HSV-1 and HSV-2* *in vitro;* Plaque reduction assay-pre-, post-; 48 h (HSV-1), 96 h (HSV-2); Vero cells; Acyclovir (IC_50_: 0.64 *μ*g/mL (HSV-1), 0.80 *μ*g/mL (HSV-2))	(1) monogalactosyl diglyceride (MGDG) (2) digalactosyl diglyceride (DGDG)	Pre: Exhibited < 50% protective effect Post: Exhibited 100% protective effect Post-HSV-1: IC_50_: 36.00 *μ*g/mL (MGDG), 40.00 *μ*g/mL (DGDG), HSV-2: IC_50_: 41.00 *μ*g/mL (MGDG), 43.20 *μ*g/mL (DGDG)	[56]
*Anti-dengue virus assay* *in vitro*; DV2 strain 16681; Real time-PCR, immunofluorescence assay; direct, pre-, post-; 5 d; Dextran sulfate (pre-), Ribavirin (post-)	(1) 13^2^-hydroxy-(13^2^-*S*)-chlorophyll b (2) phaeophorbide A (3) 13^2^-hydroxy-(13^2^-*S*)-phaeophytin b (4) purpurin 18 phytyl ester	Compound 2 inhibit dengue viral 2 replication in direct and post- stages, other compounds ineffective in all stages.	[108]

(e) Anti-bacterial activity			

*in vitro*; *S. aureus, S. typhimurium*; (1) Disc diffusion assay, (2) Microdilution assay; 10 mg/mL; Erythromycin (10–50 *μ*g/*μ*L)	(1) Stigmasterol-*β*-*D*-glucoside (2) 3-amino-4,5-dihydroxyfuran-2(3H)-one	Compound 1: Ineffective Compound 2 on *S. aureus: *18.33 mm inhibition value, MIC: 0.3125 mg/mL Compound 2 on *S. typhimurium*: 20.33 mm inhibition value, MIC: 0.625 mg/mL	[49]

(f) Anti-cancer activity			

*in vitro;* SGC-7901 cancer cells; MTT assay; 48 h; 50, 100 and 200 *μ*g/mL	Polysaccharide peptide complex: CNP-1-2	92.34 ± 0.94% of inhibition on cell growth at 200 *μ*g/mL in 48 h	[59]
*in vitro*; A549 cells; MTT assay; 72 h	(1) 13^2^-hydroxy-(13^2^-*S*)-chlorophyll b (2) phaeophorbide A (3) 13^2^-hydroxy-(13^2^-*S*)-phaeophytin b (4) purpurin 18 phytyl ester	CC_50_: (1) 43 *μ*g/mL, (2) 25 *μ*g/mL, (3) 50 *μ*g/mL, (4) 50 *μ*g/mL	[108]

(g) Cytotoxicity assay			

*in vitro*; CVS assay; 72 h; Vero cells	(1) 13^2^-hydroxy-(13^2^-R)-phaeophytin b (2) 13^2^-hydroxy-(13^2^-S)-phaeophytin a (3) 13^2^-hydroxy-(13^2^-R)-phaeophytin a	Maximum concentration that is not toxic to Vero cell is: Compound 1 (5.89 *μ*M), 2 (6.21 *μ*M), 3 (6.21 *μ*M)	[107]
*in vitro*; MTT assay; 48 h; Vero cells; 100–15000 *μ*g/mL	(1) monogalactosyl diglyceride (MGDG) (2) digalactosyl diglyceride (DGDG)	MGDG: CC_50_: 955.00 ± 7.00 *μ*g/mL DGDG: CC_50_: 922.00 ± 4.00 *μ*g/mL	[56]
